# Species specificity and intraspecific variation in the chemical profiles of *Heliconius* butterflies across a large geographic range

**DOI:** 10.1002/ece3.6079

**Published:** 2020-04-03

**Authors:** Kathy Darragh, Gabriela Montejo‐Kovacevich, Krzysztof M. Kozak, Colin R. Morrison, Clarisse M. E. Figueiredo, Jonathan S. Ready, Camilo Salazar, Mauricio Linares, Kelsey J. R. P. Byers, Richard M. Merrill, W. Owen McMillan, Stefan Schulz, Chris D. Jiggins

**Affiliations:** ^1^ Department of Zoology University of Cambridge Cambridge UK; ^2^ Smithsonian Tropical Research Institute Panama City Panama; ^3^ Department of Integrative Biology The University of Texas at Austin Austin TX USA; ^4^ Institute for Biological Sciences Universidade Federal do Pará Belém Brazil; ^5^ Biology Program Faculty of Natural Sciences and Mathematics Universidad del Rosario Bogota Colombia; ^6^ Division of Evolutionary Biology Faculty of Biology Ludwig‐Maximilians‐Universität München Munich Germany; ^7^ Institute of Organic Chemistry Technische Universität Braunschweig Braunschweig Germany

**Keywords:** chemical ecology, Lepidoptera, mate choice, pheromones, reproductive isolation, signaling

## Abstract

In many animals, mate choice is important for the maintenance of reproductive isolation between species. Traits important for mate choice and behavioral isolation are predicted to be under strong stabilizing selection within species; however, such traits can also exhibit variation at the population level driven by neutral and adaptive evolutionary processes. Here, we describe patterns of divergence among androconial and genital chemical profiles at inter‐ and intraspecific levels in mimetic *Heliconius* butterflies. Most variation in chemical bouquets was found between species, but there were also quantitative differences at the population level. We found a strong correlation between interspecific chemical and genetic divergence, but this correlation varied in intraspecific comparisons. We identified “indicator” compounds characteristic of particular species that included compounds already known to elicit a behavioral response, suggesting an approach for identification of candidate compounds for future behavioral studies in novel systems. Overall, the strong signal of species identity suggests a role for these compounds in species recognition, but with additional potentially neutral variation at the population level*.*

## Graphical abstract

In this study, we survey both inter‐ and intraspecific variation of both androconial and genital chemical profiles of male *Heliconius* across the Neotropics. We find that most variation in our samples is explained by species, with species‐specific compounds found consistently across a large geographic range, with additional, mainly quantitative, intraspecific variation. Overall, the strong signal of species identity suggests a role for these compounds in species recognition, but with additional neutral variation at the population level.

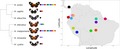

## 1 INTRODUCTION

Reproductive isolation between lineages is important for the maintenance of species diversity (Coyne & Orr, 2004). In many animals, mate choice provides a strong premating barrier, maintaining reproductive isolation (Friberg et al., 2008; Gray & Cade, 2000; Martin & Mendelson, 2016; Nagel & Schluter, 1998; Ready et al., 2006; Seehausen et al., 2008; Selz, Pierotti, Maan, Schmid, & Seehausen, 2014). Closely related species often differ in traits important for mate choice, with individuals displaying a preference for conspecific phenotypes (Jiggins, Naisbit, Coe, & Mallet, 2001; Mas & Jallon, 2005; Ryan & Guerra, 2014; Saveer et al., 2014; Yildizhan et al., 2009). These traits are predicted to show strong species‐specific differences (Gerhardt, 1982), and typically should be subject to stabilizing selection which can act to decrease intraspecific phenotypic variation (Butlin, Hewitt, & Webb, 1985; Pfennig, 1998; Ptacek, 2000). As a consequence, we would expect to find little trait variability, or at least certain features to be invariant, across species geographic ranges (Benedict & Bowie, 2009; Ferreira & Ferguson, 2002; McPeek, Symes, Zong, & McPeek, 2011; Weber, Mitko, Eltz, & Ramírez, 2016). However, these traits can also exhibit variation both within and between populations of the same species, due to either genetic drift or varying selective regimes across their ranges (Bolnick & Kirkpatrick, 2012; Ryan & Guerra, 2014; Ryan & Rand, 1993; Ryan, Rand, & Weigt, 1996).

Signals important for behavioral isolation could arise from the divergence of traits used in intraspecific communication between populations (Johansson & Jones, 2007; Mendelson & Shaw, 2012; Ryan & Rand, 1993; Smadja & Butlin, 2008). Signal divergence can be driven by various factors, both neutral and adaptive, usually involving multiple evolutionary forces (Leonhardt, Rasmussen, & Schmitt, 2013; Sun et al., 2013). A positive correlation between genetic distance and phenotypic variation is consistent with stochastic processes, such as genetic drift, playing a prominent role (Irwin, Thimgan, & Irwin, 2008). In contrast, a lack of correlation between phenotypic and genetic divergence may suggest that selection is shaping the phenotypic variation, perhaps driving divergence in different directions in each population (Campbell et al., 2010; Conrad, Paxton, Assum, & Ayasse, 2018; Hankison & Ptacek, 2008; Mullen, Vignieri, Gore, & Hoekstra, 2009).

Chemical compounds, such as sex pheromones, mediate intraspecific communication in many systems (Wyatt, 2003, 2014). The role of chemical signaling in behavioral isolation is also well established, especially among moth species (Löfstedt, 1993; Smadja & Butlin, 2008). Pheromone evolution requires changes in both the detection of pheromone by the receiver and the production of pheromone by the sender. Due to this coordination between detection and production, these pheromone blends are traditionally regarded as being under stabilizing selection toward a species stereotype (Löfstedt, 1993). Nonetheless, even when species‐specific characteristics are present, chemical composition can exhibit intraspecific variation, with both qualitative and quantitative differences found across a species range (Carde & Allison, 2016).

Studies of *Heliconius* butterflies have contributed to our understanding of adaptation and speciation (Jiggins, 2008, 2017; Merrill et al., 2015). Despite the reliance of this group on visual cues for mating (Bybee et al., 2012; Finkbeiner, Fishman, Osorio, & Briscoe, 2017; Jiggins et al., 2001; Merrill, Chia, & Nadeau, 2014; Sánchez et al., 2015), it has long been suggested that male pheromones also play a role in premating barriers (Jiggins, 2008; Merrill et al., 2015), but so far, only a few species have been studied. Behavioral experiments reveal that chemical signaling in *Heliconius erato, H. melpomene*, and *H. timareta* is important for female mate choice (Darragh et al., 2017; Mérot, Frérot, Leppik, & Joron, 2015). Previous studies have shown that *Heliconius cydno* and *H. melpomene* respond to both con‐ and heterospecific androconial chemical bouquets (Byers et al., 2019), and have identified an individual compound that is electrophysiologically and behaviorally active. Furthermore, studies of *H. cydno*, *H. doris*, *H. hecale*, *H. ismenius*, *H. melpomene*, *H. pardalinus*, *H. sara*, and *H. timareta* from Panama, Colombia, Ecuador, and Peru found that major compounds differ between species (Mann et al., 2017; Mérot et al., 2015), suggesting a potential role in reproductive isolation.

The role of chemical signaling is likely to be especially important in comimics, where visual signals alone are not sufficient to identify conspecifics (Estrada & Jiggins, 2008; Giraldo, Salazar, Jiggins, Bermingham, & Linares, 2008; Mérot et al., 2013; Sánchez et al., 2015). In contrast, chemical compounds could be part of a multimodal aposematic warning signal (Rojas et al., 2018; Rothschild, 1961), with some tentative evidence that comimics exhibit similar chemical bouquets to aid recognition by predators (Mann et al., 2017).

Here, we describe the chemical profiles of seven species of *Heliconius* from over 250 individuals collected across the Neotropics. We focus on the comimetic species *H. melpomene* and *H. erato* that are distributed widely across the Neotropics and analyzed both wing androconial and genital compounds of male butterflies. We hypothesize that compounds found consistently across the geographic range of a species are likely to be behaviorally active compounds, important for mate choice. We use *H. melpomene* as a test species due to the availability of behavioral and electrophysiological data to investigate this approach, by evaluating consistency in compound blends across different localities.

The extensive dataset analyzed here allows us to test evolutionary hypotheses, as well as identifying interesting candidate compounds for future behavioral studies. As well as interspecific variation, we also investigated intraspecific variation in chemical profiles of *H. melpomene* and *H. erato.* In both inter‐ and intraspecific datasets, we correlated chemical profile data with both geographic and genetic distances. Furthermore, to investigate if the chemical compounds are part of the aposematic comimicry signal, we sampled two different mimicry rings in western Ecuador and Panama.

## 2 MATERIALS AND METHODS

### 2.1 Sampling

**Figure 1:**
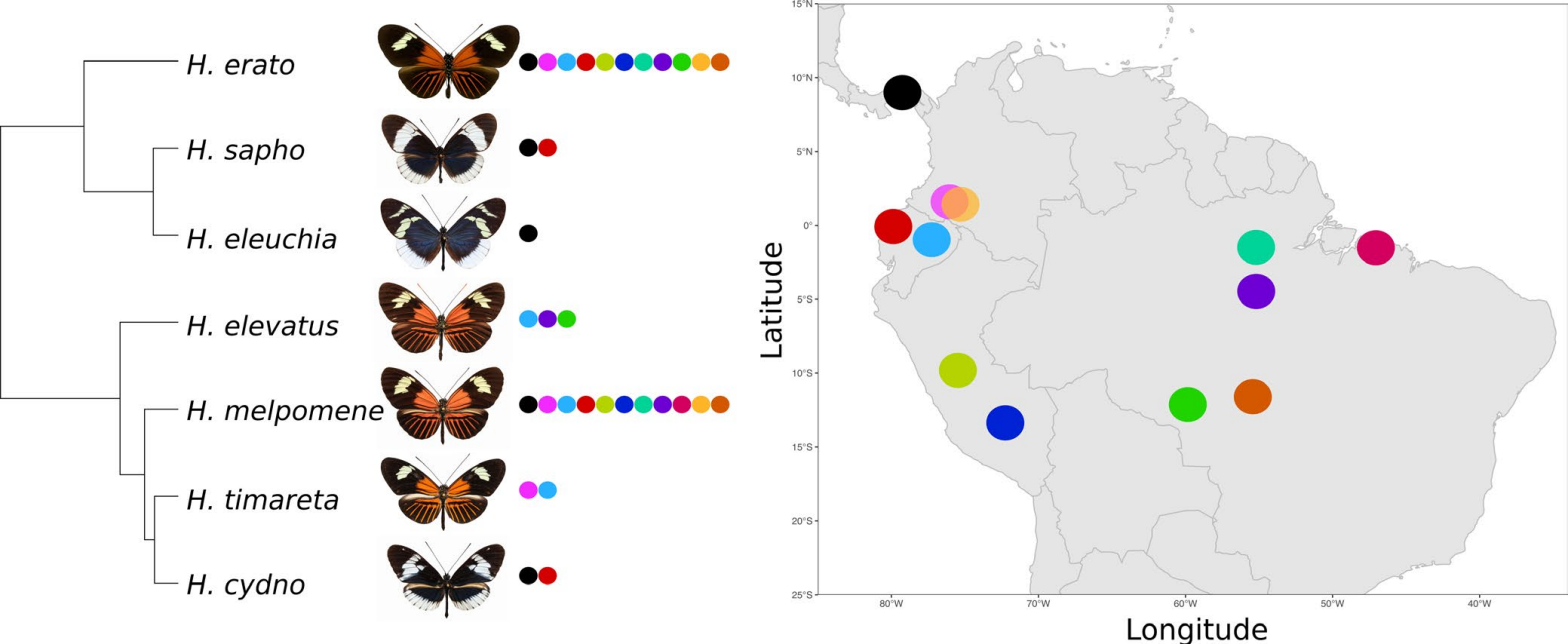
Map indicating species collected from twelve localities across the Neotropics. See Table A1 in Appendix 1 for sample numbers. The phylogeny was previously published by Kozak et al. (2015)

Between February 2016 and August 2017, wild males of *Heliconius cydno*, *H. elevatus*, *H. eleuchia*, *H. erato*, *H. melpomene*, *H. sapho*, and *H. timareta* were collected with hand nets from twelve localities. Between two and fifteen males were chemically analyzed per population (Figure 1, Table A1 in Appendix 1), and one representative from each subspecies of *H. erato* and *H. melpomene* was used for whole‐genome sequencing (Table A2 in Appendix 1). We follow the latest *Heliconius* taxonomy (Lamas & Jiggins, 2017).

### 2.2 Extraction and chemical analysis of tissues

The androconial region of the wing, previously described as the gray–brown overlapping region of the hind wing (Darragh et al., 2017), as well as the genitalia, was dissected for analysis immediately after collection. For chemical extraction, the tissue was soaked in 200 μl dichloromethane containing 200 ng 2‐tetradecyl acetate (internal standard) in 2‐ml glass vials with PTFE‐coated caps (Agilent) for one hour. The solvent was then transferred to new vials, maintained cool in the field, and stored at −20°C upon return. Androconial samples were evaporated to a reduced volume at room temperature prior to analysis. Extracts were analyzed by GC/MS using an Agilent model 5977 mass‐selective detector connected to an Agilent GC model 7890B and equipped with an Agilent ALS 7693 autosampler. HP‐5MS fused silica capillary columns (Agilent, 30 m × 0.25 mm, 0.25 µm) were used. Injection was performed in splitless mode (250°C injector temperature) with helium as the carrier gas (constant flow of 1.2 ml/min). The temperature program started at 50°C, was held for 5 min, and then rose at a rate of 5°C/min to 320°C, before being held at 320°C for 5 min. Components were identified by comparison of mass spectra and gas chromatographic retention index with those of authentic reference samples and also by analysis of mass spectra. Components were quantified using 2‐tetradecyl acetate as an internal standard. Only compounds eluting earlier than hexacosane were analyzed in androconial samples and those earlier than nonacosane in genital samples (Darragh et al., 2017). We globally removed compounds that were not found in at least half of all individuals from a given population.

### 2.3 DNA extraction and library preparation

We used a representative individual from each subspecies of *H. erato* and *H. melpomene* from across their range. Individuals were genotyped with medium‐ to high‐coverage whole‐genome sequencing. We used two sequencing approaches. Genomic DNA of individuals whose ID starts with SR or KK (C. Kozak collection, *n* = 14) was extracted from thorax tissue with Qiagen MagAttract beads. The KAPA Biosystems kit was used to prepare paired‐end 2 × 150 base pair libraries with inserts of 50–200 bp after SPRI size selection. Libraries were quality‐controlled using the Agilent 2100 Bioanalyzer and indexed with the KAPA Single‐Indexed Adapter Kit. Libraries were sequenced on the Illumina HiSeq 4000 platform by Novogene, Tianjin, People's Republic of China. For the remaining newly sequenced individuals (*n* = 6, starting with CAM or 14N, Cambridge and N. Nadeau collections, respectively), we extracted genomic DNA with Qiagen DNeasy kits (Qiagen) from thorax tissue. The libraries for these individuals were TruSeq Nano, gel‐free libraries and were sequenced on Illumina HiSeq 2500 platform (v4 chemistry) by Novogene (Hong Kong). Whole genomes for four individuals were obtained from public databases. Accession numbers and individual information can be found in Table A2 in Appendix 1.

### 2.4 Calculation of genetic and geographic distance matrices

To explore genetic distance among the studied *H. erato* (*n* = 12) and *H. melpomene* (*n* = 13) populations, we computed whole‐genome genetic covariance matrices and performed MDS for each species separately. A whole‐genome sequence from a representative individual from each population was used (Table A2 in Appendix 1). Genotypes were inferred from reads mapped to the *H. melpomene* (v2.5) and *H. erato demophoon* genome scaffolds (Challis, Kumar, Dasmahapatra, Jiggins, & Blaxter, 2016; Davey et al., 2017; Heliconius Genome Consortium, 2012; Van Belleghem et al., 2017) with bwa v0.7.15 (Li & Durbin, 2009). We computed a whole‐genome pairwise identical‐by‐state (IBS) matrix with a random sampled read from each position in the genome, implemented in ANGSD v0.912 (Korneliussen, Albrechtsen, & Nielsen, 2014) (angsd ‐bam bam.path.list ‐minMapQ 30 ‐minQ 20 ‐GL 2 ‐doMajorMinor 1 ‐doMaf 1 ‐SNP_pval 2e‐6 ‐doIBS 1 ‐doCounts 1 ‐doCov 1 ‐makeMatrix 1 ‐minMaf 0.05).

An interspecific genetic distance matrix was constructed using the function “cophenetic.phylo” from the *ape* package (Paradis & Schliep, 2018) with a previously published phylogeny (Kozak et al., 2015). Geographic distance matrices were created by inputting the coordinates of collection localities into the function “distm” in the *geosphere* package to calculate the Haversine great‐circle distance between points (Hijmans, 2017).

### 2.5 Statistical analyses

#### 2.5.1 Inter‐ and intraspecific indicator compounds

We carried out indicator analysis using the *indicspecies* package (Cáceres & Legendre, 2009). Groupings are decided a priori (in this case, species or population), and compounds are determined which act as indicators of these groups. The best indicators are those which are only found in a single group (specificity), and all group members possess the compound (coverage); such a compound would have an indicator value of 1. The specificity of a compound is calculated based on the amount of compound found in each individual, while the coverage considers only presence or absence of the compound. We used the function “indicators” to investigate both which single compounds and which combinations of compounds best predict group membership. We used the function “pruneindicators” to find the single compounds or combinations of compounds which had the highest indicator values.

#### 2.5.2 Variation in chemical profiles

Divergence in chemical profiles across species and populations was estimated with nonmetric multidimensional scaling (NMDS) ordination in three dimensions, based on a Bray–Curtis similarity matrix using absolute peak areas. We used the “metaMDS” function in the *vegan* package version 2.5‐1 (Oksanen et al., 2017) and visualized the NMDS using the *ade4* package (Dray & Dufour, 2007).

We assessed the relative importance of relevant factors in driving the variation in chemical profiles with multivariate statistical analyses. These factors included species identity, geographic region, and individual locality. We excluded subspecies as a factor because, in *Heliconius*, these are determined based on their, sometimes very subtle, difference in wing color pattern, with extensive gene flow across the genome between subspecies (Van Belleghem et al., 2017). It is therefore more biologically relevant to include locality in the model, to account for genetic drift between subspecies, and since locality and subspecies are highly correlated, we cannot include both. To compare overall variation in chemical composition between groups, we carried out PERMANOVA (permutational multivariate analysis of variance) testing based on a Bray–Curtis distance matrix, using the “adonis2” function in the *vegan* package with 1,000 permutations. We investigated each term in the model sequentially, starting with species identity, the main clustering factor found from visualization with NMDS, followed by geographic region (Panama vs. Western Andes vs. Eastern Andes vs. Amazon), and finally individual collecting localities. Model goodness of fit was evaluated by Akaike's information criterion (AIC). In general, we chose the model with the lowest AIC value; however, if two models were within two AIC of each other, we chose the simplest model as the best fit (Table A3 in Appendix 2). We followed these PERMANOVA tests with *post hoc* pairwise testing using the function “pairwise.perm.MANOVA” in the *RVAideMemoire* package, with Bonferroni correction, to identify which grouping factors were significantly different (Hervé, 2018). We repeated the PERMANOVA within species, in *H. erato* and *H. melpomene,* to investigate fine‐scale intraspecific geographic patterns. In the within‐species analysis, we included geographic region (Panama vs. Western Andes vs. Eastern Andes vs. Amazon) and individual collecting localities as the two factors.

One issue with distance‐based analyses such as PERMANOVA is that differences in dispersion between groups can be confounded with differences in location (Warton, Wright, & Wang, 2012). To confirm these analyses and account for this issue, we implemented multivariate generalized linear models using the function “ManyGLM” from the *mvabund* package (Wang, Naumann, Wright, & Warton, 2012). We modeled the data using a negative binomial distribution, which we found to be appropriate through examination of residual plots. For interspecific analyses, we included species, region, and locality nested within region in the model. For intraspecific analyses, we included region and locality nested within region. The “ManyGLM” function fits models to each chemical compound, summing the test statistics to give a multivariate test statistic known as Sum‐of‐LR. This statistic can be tested for significance using resampling methods. We carried out backward elimination and compared the fit of models by using the “ANOVA.manyglm” function with a likelihood ratio test (Table A4 in Appendix 2). We can also determine which compounds are driving between‐group differences by looking at the individual contribution of each compound to the Sum‐of‐LR, with *p*‐values adjusted for multiple testing using the “adjust” option.

### 2.6 Phylogenetic and geographic distance

Shared ancestry can explain part of the variation in a species’ chemical profile. Using the interspecific genetic distance matrix calculated above, we tested for a correlation between phylogenetic distance and chemical profile divergence. We carried out partial Mantel tests, controlling for geographic distance, using the *vegan* package (Oksanen et al., 2017). To investigate the role of geographic distance in chemical profile divergence, we compared geographic and chemical distances matrices, controlling for genetic distance, with partial Mantel tests. To visualize the species phylogeny (Kozak et al., 2015), we used the “plot.phylo” function from the ape package (Paradis & Schliep, 2018).

### 2.7 Genomic and chemical distance within species

We calculated intraspecific genetic distances using genome sequences from 11 *H. erato* and 13 *H. melpomene* populations. We visualized genetic distances in two dimensions using MDS with the function “cmdscale.” We tested for a correlation between intraspecific genetic distance and chemical profile divergence with partial Mantel tests, controlling for geographic distance, using the *vegan* package (Oksanen et al., 2017). Hybrids between populations of the same species were excluded from this analysis (Table A2 in Appendix 1). We also used partial Mantel tests to investigate the role of geographic distance, while controlling for genetic distance.

### 2.8 Comimics and similarity of chemical profiles

We used samples of two mimicry rings from two localities, Panama and western Ecuador. *H. melpomene* and *H. erato* form one mimicry ring, while *H. cydno* and *H. sapho* form another, with the addition of *H. eleuchia* in western Ecuador (Figure 1). We visualized these samples but did not carry out statistical analyses due to the pseudoreplication caused by the similarity of individuals within a species. More species comparisons would be needed for further analysis.

All statistical analyses were performed with *R* version 3.5.1 (R Core Team, 2018). Figures were made using a palette of colors optimized for color blindness (Wong, 2011). We used ggplot2 for violin and boxplots (Wickham, 2009). Sequencing data are available from ENA under accession number PRJEB35570. GCMS chromatograms, other data, and R scripts used for analysis are available from Open Science Framework (OSF) (https://osf.io/28yfk/).

## 3 RESULTS

### 3.1 Chemical compounds in androconia and genitals

We sampled 252 androconia and 275 genitals across 42 populations of seven species and identified 349 compounds in the genitals and 157 in the androconia (Tables S1 and S2). Of the total number of androconial compounds, 38% are fatty acid derivatives, 20% aromatics, 10% terpenoids, 1% macrolides, <1% lactones, and 31% unknown or unidentified compounds. Of the genital compounds, 17% are fatty acid derivatives, 7% aromatics, 10% terpenoids, 1% lactones, 12% macrolides, and 44% unknown or unidentified compounds. The main difference is that there are more macrolides in the genitals than in androconia.

**Figure 2:**
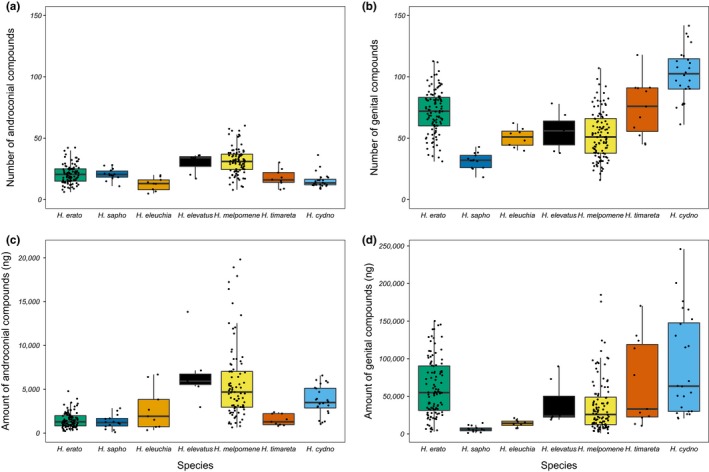
Composition of androconial and genital chemical bouquets across seven *Heliconius* species. Species significantly differ in (a) number of androconial compounds (ANOVA, *F*_6,245_ = 21.54, *p* < .001), (b) number of genital compounds (ANOVA, *F*_6,268_ = 36.15, *p* < .001), (c) amount of androconial compounds (ANOVA, *F*_6,245_ = 11.55, *p* < .001), and (d) amount of genital compounds (ANOVA, *F*_6,268_ = 11.62, *p* < .001). Four outlier individuals were removed from c

*Heliconius* species varied considerably in the amount and number of compounds (Figure 2). Between species, there was variation in the number of compounds per individual and the overall amount of compounds detected (Tables S1 and S2). For the androconia, *H. eleuchia* had the fewest compounds (13 ± 5) and *H. melpomene* the highest (32 ± 11) (mean ± standard deviation). *H. sapho* had the lowest total amount of androconial compounds at 1,300 ± 803 ng and *H. melpomene* the highest at 7,254 ± 8,242 ng. The species with the fewest genital compounds was *H. sapho* with 32 ± 7 and the highest *H. cydno* with 102 ± 21. *H. sapho* also had the lowest total amount of genital compounds at 6,642 ± 3,975 ng and *H. cydno* the highest at 91,167 ± 67,122 ng. These values are within the same order of magnitude as expected from previous work on male sex pheromones in the butterfly *Bicyclus anynana* (van Bergen, Brakefield, Heuskin, Zwaan, & Nieberding, 2013; Nieberding et al., 2012). Using *H. erato* as an example, the androconial bouquet is 0.00002% and genital bouquet 0.0007% of total body weight (Montgomery, Merrill, & Ott, 2016). In general, a higher number of compounds and total amount of compounds are found in the genitals than in the androconial patches of *Heliconius* wings.

### 3.2 Are there species‐specific chemical compounds?

In order to identify candidate species recognition pheromones, we examined our data for species‐specific compounds using indicator analysis. In most species that we examined, there were single androconial compounds that were strong indicators of species identity (Table 1). For example, geranylgeranylacetone was found only in *H. erato* and was consistently present across samples of this species. Similarly, octadecanal, a compound previously shown to be electrophysiologically and behaviorally active (Byers et al., 2019), was found almost exclusively in *H. melpomene* (specificity = 0.999). *H. cydno* and *H. eleuchia* had the weakest indicator scores—in *H. cydno* because the best indicator compound was not found in all individuals examined (coverage = 0.667), and in *H. eleuchia* because the best indicator compound was also found in other species (specificity = 0.747). There were similarly species‐specific genital compounds in all species except *H. sapho* and *H. timareta*, where a combination of two compounds was the best predictor (Table 2). Similar to the androconia, in *H. melpomene*, the best indicator compound for genitalia has known behavioral activity, in this case the anti‐aphrodisiac, (*E*)‐β‐ocimene (Schulz, Estrada, Yildizham, Boppré, & Gilbert, 2008). For *H. erato*, we identified a terpene ester which is only found in *H. erato* individuals and no other species. Other terpene esters were also almost perfect indicator compounds of *H. erato*.

**Table 1:** Androconial compounds which are the best indicators of species identity

Species/compound	A: specificity	B: coverage	sqrtIV
*Heliconius cydno*			
Unknown aromatic (RI = 2,130)	1	0.667	0.816
*H. eleuchia*			
Hexahydrofarnesyl acetone	0.747	1	0.864
*H. elevatus*			
Homovanillyl alcohol	0.912	1	0.955
*H. erato*			
Geranylgeranylacetone	1	1	1
*H. melpomene*			
Octadecanal	0.999	1	1
*H. sapho*			
Methyl 4‐hydroxy−3‐methoxybenzoate	0.866	1	0.931
*H. timareta*			
5‐Decanolide	1	0.889	0.943

Note

A is a measure of species specificity of the compounds, B is a measure of species coverage, and sqrtIV is the indicator value which considers both A and B and ranges from 0 (compound not present in any individuals of that species) to 1 (compound only present in that species, and present in all individuals).

**Table 2:** Genital compounds which are the best indicators of species identity. A, B, and sqrtIV as in Table 1

Species/compound	A: specificity	B: coverage	sqrtIV
*Heliconius cydno*			
Unknown ester (RI = 1,390)	0.999	1	0.999
*H. eleuchia*			
Unknown macrolide (RI = 1,878)	0.969	1	0.984
*H. elevatus*			
Icosenol	0.908	1	0.953
*H. erato*			
Unknown terpene ester (RI = 2,494)	1	1	1
*H. melpomene*			
(*E*)‐β‐Ocimene	0.865	1	0.930
*H. sapho*			
(*Z*)‐3‐Hexenyl isobutyrate and unknown (RI = 1,691)	0.957	0.923	0.940
*H. timareta*			
Butyl oleate and (*Z*)−9‐octadecen−13‐olide	0.915	1	0.956

### 3.3 What factors affect interspecific variation in chemical profiles?

**Figure 3:**
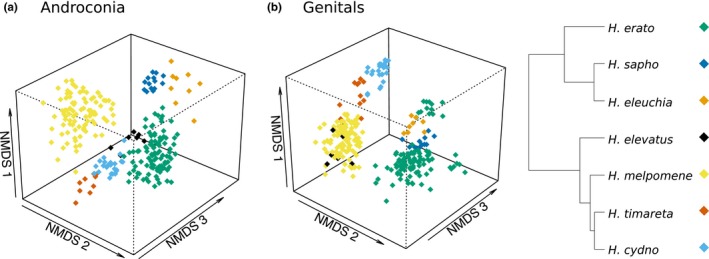
NMDS (nonmetric multidimensional scaling) plot illustrating in three dimensions the variation in chemical compounds of male *Heliconius* of different species. (a) Androconial compound bouquets differ significantly between species. Stress = 0.155. (b) Genital bouquets also differ significantly between species. Stress = 0.121

Our sampling allowed us to investigate how variation in chemical composition is partitioned within and between species, and determine the extent to which chemistry is a species‐diagnostic trait. Visualization of the chemical profiles reveals that individuals mostly group by species for both androconial and genital chemical bouquets (Figure 3). Species significantly differ in their androconial bouquet, with species identity accounting for 58% of the overall variation in chemical profiles (PERMANOVA, Species, *F*_6,251_ = 72.16, *p* < .001). All pairwise comparisons of species are significantly different (Table A5 in Appendix 3). A further 4% of variation can be explained by region (Amazon/Eastern Andes/Western Andes/Panama), and 3% by locality nested within region (PERMANOVA, Region, *F*_3,251_ = 9.96, *p* < .001; Region/Locality, *F*_8,251_ = 2.65, *p* < .001). Finally, 4% of variation is explained by an interaction between species and region (PERMANOVA, Species*Region, *F*_6,251_ = 4.82, *p* < .001).

**Figure A1:**
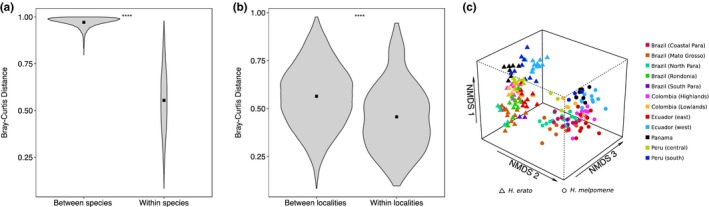
Pairwise androconial Bray–Curtis distances between individuals of *H. erato* and *H. melpomene* (a) between and within species and (b) between and within localities of the same species. (c) NMDS (nonmetric multidimensional scaling) plot illustrating the variation in androconial chemical compounds of male *H. erato* and *H. melpomene* from different localities

**Figure A2:**
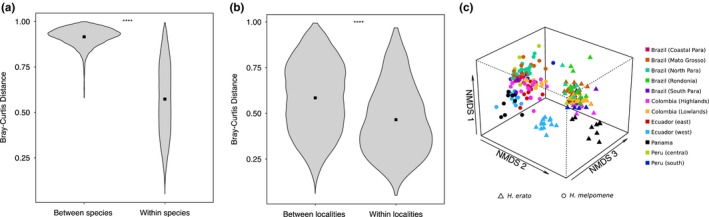
Pairwise genital Bray–Curtis distances between individuals of *H. erato* and *H. melpomene* (a) between and within species and (b) between and within localities of the same species. (c) NMDS (nonmetric multidimensional scaling) plot illustrating the variation in genital chemical compounds of male *H. erato* and *H. melpomene* from different localities

The results were similar for the genital bouquets, with species identity accounting for 51% of the variation in chemical profiles (PERMANOVA, Species, *F*_6,274_ = 59.81, *p* < .001). All pairwise comparisons are significant apart from *H. elevatus* and *H. melpomene* (Table A6 in Appendix 3). A further 5% of variation can be explained by region (Amazon/Eastern Andes/Western Andes/Panama), and 3% by locality nested within region (PERMANOVA, Region, *F*_3,274_ = 12.43, *p* < .001; Region/Locality, *F*_8,274_ = 2.92, *p* < .001). Finally, 6% of variation is explained by an interaction between species and region (PERMANOVA, Species*Region, *F*_6,274_ = 6.52, *p* < .001). For both androconial and genital chemical profiles, most variation is explained by species identity, rather than geographic location, as confirmed by ManyGLM (Tables A7 and A8 in Appendix 3). We also confirmed this by comparison of within and between species and locality Bray–Curtis distances (Figure A1 and Figure A2, Appendix 4).

### 3.4 Does phylogenetic distance explain chemical profile divergence?

Using whole‐genome sequence data, we explored the degree to which variation between species can be explained by geographic and genetic distance among the samples. We carried out partial Mantel tests to investigate the correlation between two variables while controlling for a third variable. When controlling for geographic distance, genetic divergence is strongly correlated with both androconial and genital chemical divergence (partial Mantel test, androconia, *r* = .7871, *p* = .001; genitals, *r* = .6936, *p* = .001). When controlling for genetic distance, geographic distance is significantly but weakly correlated with androconial and genital chemical divergence (partial Mantel test, androconia, *r* = .072, *p* = .001; genitals, *r* = .046, *p* = .007).

### 3.5 Do we find population‐specific chemical compounds?

We used an indicator analysis to search for compounds unique to specific populations of *H. erato* and *H. melpomene*. Most intraspecific differences are due to quantitative rather than qualitative differences between populations, perhaps explaining why many population indicators were weak as they are also found in other regions at different amounts (Tables A9 and A10 in Appendix 3). The only exception is *H. e. cyrbia* (western Ecuador) that has many genital compounds unique to this region (Table A9 in Appendix 3).

### 3.6 What factors affect intraspecific variation in chemical profiles of *H. erato* and *H. melpomene*?

**Figure 4:**
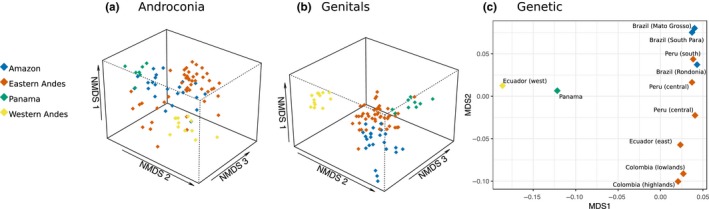
Plots of androconial, genital, and genetic distance between *Heliconius erato* populations. (a) NMDS (nonmetric multidimensional scaling) plot illustrating in three dimensions the variation in androconial chemical compounds. Stress = 0.174. (b) NMDS plot illustrating in three dimensions the variation in genital chemical compounds. Stress = 0.118. (c) MDS plot illustrating in two dimensions the genetic distance between populations of *H. erato*

We also wanted to determine the sources of variation within species using our broad sampling of populations across the ranges of *H. erato* and *H. melpomene*. For *H. erato*, there was a strong grouping of individuals by region (Figure 4), with 27% of variation in androconial profiles being explained by region and 11% by locality nested within region (PERMANOVA, Region, *F*_3,87_ = 11.16, *p* < .001; Locality, *F*_6,87_ = 2.35, *p* < .001). All four regions are significantly different from each other (pairwise permutation MANOVAs, *p* < .01). For *H. erato* genital compounds, 37% of variation is explained by region and 11% by locality nested within region (PERMANOVA, Region, *F*_3,91_ = 19.01, *p* < .001; Locality, *F*_6,91_ = 2.83, *p* < .01). All four regions are significantly different from each other (pairwise permutation MANOVAs, *p* < .05).

**Figure 5:**
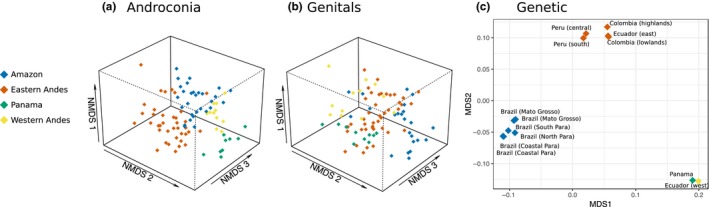
Plots of androconial, genital, and genetic distance between *Heliconius melpomene* populations. (a) NMDS (nonmetric multidimensional scaling) plot illustrating in three dimensions the variation in androconial chemical compounds. Stress = 0.151. (b) NMDS plot illustrating in three dimensions the variation in genital chemical compounds. Stress = 0.161. (c) MDS plot illustrating in two dimensions the genetic distance between populations of *H. melpomene*

These geographic differences in chemical profiles are not as strong in *H. melpomene* (Figure 5). For *H. melpomene* androconial compounds, the best model only includes region, not locality, with 18% of variation explained by region (PERMANOVA, Region, *F*_3,86_ = 6.73, *p* < .01). The West Andes population (*H. m. cythera*) is not significantly different from either East Andes (multiple populations) or Panama (*H. m. rosina*) (pairwise permutation MANOVAs, *p* = .072); however, the other comparisons are significantly different (pairwise permutation MANOVAs, *p* < .05). For *H. melpomene* genital compounds, 20% of variation is explained by region and 12% by locality nested within region (PERMANOVA, Region, *F*_3,103_ = 8.91, *p* < .001; Locality, *F*_7,103_ = 2.34, *p* < .001). All regions are significantly different from each other (pairwise permutation MANOVAs, *p* < .05), apart from West Andes and Amazon (pairwise permutation MANOVAs, *p* = .120). Both species show variation between geographic locations, with more variance explained by region in *H. erato* than *H. melpomene*. These results were confirmed by ManyGLM tests (Tables A11, A12, A13, A14 in Appendix 3).

### 3.7 Does genetic distance explain chemical divergence in *H. erato* and* H. melpomene*?

In *H. erato*, chemical distance is positively correlated with genetic distance, when accounting for geographic distance, although this correlation is weak for androconia (partial Mantel test, androconia, *R* = .164, *p* = .001; genitals, *R* = .348, *p* = .001). When we account for genetic distance, geographic distance is weakly correlated with androconial chemical distance and not correlated with genital chemical distance (partial Mantel test, androconia, *R* = .151, *p* = .002; genitals, *R* = −.0775, *p* = .966).

*Heliconius melpomene* genitals show similar patterns to *H. erato*, but variation in the androconia is explained by geographic but not genetic distance. When accounting for geography, genetic divergence is not correlated with androconial chemical divergence and is correlated only weakly with genital chemical divergence (partial Mantel test, androconia, *R* = .02874, *p* = .141, genitals, *R* = .1203, *p* = .001). When we first consider genetic distance, geographic distance is weakly positively correlated with androconial chemical distance, but not genital chemical distance (partial Mantel test, androconia, *R* = .1795, *p* = .002; genitals, *R* = −.004, *p* = .563).

### 3.8 Is there evidence for similarity between comimics in chemical profile?

**Figure 6:**
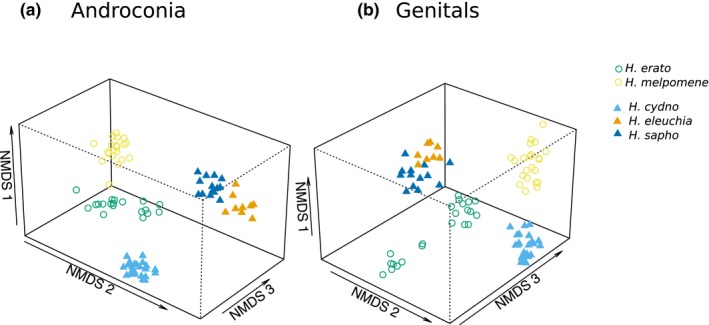
NMDS (nonmetric multidimensional scaling) plot illustrating in three dimensions the variation in chemical compounds of male *Heliconius* from Panama and western Ecuador. *H. erato* and *H. melpomene* are comimics (circles), while *H. cydno*, *H. eleuchia*, and *H. sapho* form a second comimicry group (triangles). (a) Androconial chemical bouquets. Stress = 0.098. (b) Genital chemical bouquets. Stress = 0.094

We investigated the effect of mimicry ring on chemical profile using individuals collected in Panama and western Ecuador from two mimicry rings (Figure 6). Consistent with our interspecific analyses, we find that species is the main determinant of androconial and genital bouquets. *H. sapho* and *H. eleuchia* group closely in the NMDS visualization; however, they are closely related and so it is unclear whether this similarity is due to comimicry or shared ancestry. Especially for the androconia, *H. erato* and *H. melpomene* seem to be more similar than we might expect given their phylogenetic distance.

All the results described above show a consistent pattern when unidentified compounds were not included in the analysis (Appendix 5). Interspecific analyses were also consistent when repeated without populations with a sample of fewer than five individuals (this removed seven populations from androconial analysis and five from genital analysis) (Appendix 5).

## 4 DISCUSSION

*Heliconius* butterflies represent a continental‐scale adaptive radiation (Kozak et al., 2015). Speciation in this group is often associated with divergence in wing color pattern, and pattern variation plays an important role in speciation and mate preference (Jiggins, 2008; Jiggins et al., 2001; Merrill et al., 2011, 2015, 2019; Sánchez et al., 2015). However, one of the surprising findings to emerge from comparative genomic analysis is the wealth of chemosensory genes (Heliconius Genome Consortium, 2012), suggesting that chemical signaling may play an important role in the biology of the system, such as host plant choice and mate choice. To begin to understand the role of chemical signaling in this radiation, we have extensively surveyed both inter‐ and intraspecific variation of *Heliconius's* androconial and genital chemical profiles across the Neotropics. We find that most of the variation in chemical profile across our samples is explained by species, and we identify key chemicals serving as indicators for each species. Nonetheless, there is also intraspecific variation in chemical profiles. This variation is mainly quantitative in nature, with the exception of *H. erato cyrbia* which has compounds not found in other *H. erato* populations. Our results are also in agreement with the prediction of convergence between comimics, supporting an earlier hypothesis (Mann et al., 2017). Our work sets the stage for further research into the biology and function of chemical profiles, and their role in within‐ and between‐species signaling.

It would be challenging to conduct behavioral experiments on large numbers of species and populations, and therefore, identifying the behaviorally active components in pheromone blends across a radiation is beyond the scope of a single study. Other studies have also attempted to predict male sex pheromones without behavioral data, by selecting based on multiple criteria such as male specificity and abundance (Bacquet et al., 2015). This stepwise selection of candidates focuses on within‐species characteristics such as abundance, without considering the presence of the compound in other species. We hypothesized that consistent species‐specific compounds are likely to be biologically important. We present an alternative method to detect candidate pheromones by evaluating both the presence of a compound across the geographic range of a species as well as the presence of the compound in other species. This approach has multiple advantages, including simple mathematics and the ability to evaluate combinations of compounds as well as single compounds. The compounds identified in this study as indicators for the androconia and genitals of *H. melpomene*, octadecanal and (*E*)‐β‐ocimene, respectively, have both been previously shown to be behaviorally active (Byers et al., 2019; Schulz et al., 2008). Combining broad geographic sampling with indicator analysis therefore provides a promising approach to determine potential pheromone components in other species, which could be tested behaviorally. Our analyses have already identified a number of compounds that could now be tested functionally, such as the androconial compound geranylgeranylacetone in *H. erato*.

Chemical profiles are predicted to be highly species‐specific if they are involved in species recognition during mating. For instance, orchid bee chemical blends, presumably important for mating and species recognition, show high species specificity, as well as within‐species variability, which can be partly explained by geography (Brand et al., 2019; Weber et al., 2016; Zimmermann, Roubik, & Eltz, 2006). We see similar patterns in *Heliconius*, with greater interspecific than intraspecific differences in chemical profiles. The magnitude of intraspecific differences is smaller in *Heliconius*, likely due to the fact that orchid bees collect their blends from the environment (Eltz, Whitten, Roubik, & Linsenmair, 1999). In both cases, species identity is the best predictor of chemical divergence, with geographic location able to explain some intraspecific differences. One exception to this is *H. elevatus* which does not group separately from its comimic *H. melpomene* for genital compounds, despite the fact that these species are not especially closely related in the *Heliconius* phylogeny. Further samples are needed to confirm that this result is not due to the small sample of *H. elevatus* in this study. As in orchid bees, species differences are often consistent across a large geographic range, suggesting that they could be important for reproductive isolation between species (Weber et al., 2016).

We found a correlation between chemical distance and genetic distance. This suggests that neutral evolutionary forces are important in the evolution of chemical bouquets. The correlation between genital chemical distance and genetic distance is a much stronger correlation than previously reported (Estrada, Schulz, Yildizhan, & Gilbert, 2011), possibly due to the quantitative nature of our data. The strong signal of neutrality suggests that the majority of compounds in the bouquets are neutrally evolving. For example, in the genital bouquet of *H. melpomene*, one compound, (*E*)‐β‐ocimene, can act by itself as an anti‐aphrodisiac, with other components of the bouquet thought to moderate its evaporation rate (Schulz et al., 2008). In the future, focusing on the evolutionary patterns of only compounds which exhibit behavioral or electrophysiological responses, rather than the entire bouquet, may disentangle the processes involved in the evolution of these profiles.

*Heliconius erato* and *H. melpomene* both exhibit extensive color pattern variation across their geographic range (Sheppard, Turner, Brown, Benson, & Singer, 1985) and these populations also differ in their androconial and genital bouquets. While traditionally predicted to be under stabilizing selection, intraspecific variation between populations in chemical profiles has been documented in other Lepidoptera (Carde & Allison, 2016). Chemical divergence in putative male sex pheromones between populations of *Bicyclus anynana* is reported to be as large as differences between *Bicyclus* species and is greater than predicted by genetic divergence (Bacquet et al., 2016). This is in contrast to what we find here, where interspecific differences are much greater than intraspecific ones.

Interestingly, *Heliconius erato cyrbia* produces many unique genital compounds and is also the most genetically divergent *H. erato* population in our study, suggesting that genetic drift is important for the evolution of chemical profiles in *Heliconius*. Across all *H. erato* populations, we find a correlation between chemical distance and genetic distance, which is weaker for androconial bouquets. In *H. melpomene*, genetic distance is also weakly correlated with genital chemical divergence. These correlations suggest that some of the geographic variation between populations could be neutral, with stochastic processes important for bouquet evolution in *Heliconius*. In contrast, androconial chemical variation in *H. melpomene* is better explained by geographic distance. This might imply that other evolutionary forces are important for chemical profile evolution in *H. melpomene*.

One factor potentially involved in geographic variation is larval host plant use. Feeding on different host plants as a larvae affects the production of some minor components of both androconial and genital chemical bouquets (Darragh et al., 2019)*.* The major components, however, are unaffected by larval host plant, suggesting that any dietary precursors required for compound production are present in different *Passiflora* species (Darragh et al., 2019)*.* In Panama, *H. cydno* and *H. melpomene* both feed on *P. menispermifolia* (Merrill, Naisbit, Mallet, & Jiggins, 2013), and yet have different chemical profiles, highlighting that from the same precursors different species can produce different compounds. Furthermore, it is often unclear which is the major *Passiflora* host plant of any particular *Heliconius* population. The composition of *Passiflora* species varies geographically (Benson, 1978; Benson, Brown, & Gilbert, 1975), and both host preference and level of host specificity vary between populations of the same *Heliconius* species (Castro, Zagrobelny, Cardoso, & Bak, 2018). A greater understanding of the variation in larval diet of *Heliconius* across the Neotropics will help us understand how much geographic variation in chemical profile can be attributed to host plant use.

*Heliconius* butterflies are an excellent example of visual mimicry, with different species converging on the same warning color patterns (Merrill et al., 2015; Sheppard et al., 1985; Sherratt, 2008). It has been suggested that chemical compounds could also contribute to mimicry between species (Dettner & Liepert, 1994; Mann et al., 2017). In this study, we find patterns consistent with predictions of convergence between comimics. Individuals within particular comimicry groups, such as *H. melpomene* and *H. erato,* seem to converge on a more similar chemical profile. Most known examples of chemical mimicry come from systems of deception, for example, mimicry of ant alarm pheromones by rove beetles to avoid predation, rather than mimicry of aposematic warning signals (Dettner & Liepert, 1994; Stoeffler, Maier, Tolasch, & Steidle, 2007; Vereecken & McNeil, 2010). We suggest that in *Heliconius* different components of the bouquet could be important for chemical mimicry and species recognition, reducing conflict between these selection pressures.

Convergence of genital bouquets between comimics could be due to the anti‐aphrodisiac function of these compounds (Gilbert, 1976; Schulz et al., 2008). Anti‐aphrodisiac compounds are transferred from males to females during mating to deter future matings from other males. Convergence in wing pattern between comimics could result in harassment not only by conspecific but also heterospecific males (Estrada & Jiggins, 2008). The use of the same anti‐aphrodisiac by comimics could combat interspecific attraction by deterring males of both species, as highlighted by the production of (*E*)‐β‐ocimene by *H. erato* and *H. melpomene,* as well as other *Heliconius* species (Estrada et al., 2011).

Compounds could also play a role in predator deterrence. Genital compounds were originally suggested to form part of the antipredation signal (Eltringham, 1925). We detected 2‐s‐butyl‐3‐methoxypyrazine in the genitals of *H. melpomene*, *H. cydno*, and *H. timareta*, and 2‐isobutyl‐3‐methoxypyrazine in the genitals of *H. melpomene* and *H. cydno*, both compounds known to deter predators in the wood tiger moth (Burdfield‐Steel, Pakkanen, Rojas, Galarza, & Mappes, 2018; Rojas et al., 2017, 2018; Rojas, Mappes, & Burdfield‐Steel, 2019). More generally, methoxypyrazines act as warning odors in other insects (e.g., Lepidoptera, Rothschild, Moore, & Brown, 1984; fireflies, Vencl et al., 2016), effective against avian predators (Guilford, Nicol, Rothschild, & Moore, 1987). Further investigation will be required to determine if odors of *Heliconius* butterflies act as antipredation signals.

Overall, our study reveals strong species differences in bouquets and the presence of species‐specific compounds, as well as intraspecific variation. A pattern of species specificity alongside intraspecific variation could be the result of a balance between stabilizing selection toward a species stereotype, sexual selection promoting diversity, and geographic segregation alongside selection and drift. A challenge for the field is the feasibility of testing for the biological relevance of hundreds of compounds in many species, but we hope that our innovative analysis will stimulate not only further targeted functional studies of putatively important compounds, but also large chemical profile surveys in other study systems of evolutionary interest.

## CONFLICT OF INTEREST

None declared.

## AUTHOR CONTRIBUTIONS

Kathy Darragh, Gabriela Montejo‐Kovacevich, Krzysztof Kozak, Colin Morrison, Owen McMillan, Clarisse Figueiredo, Jonathan Ready, Camilo Salazar, Mauricio Linares, and Chris Jiggins collected samples. Kathy Darragh wrote the manuscript and analyzed the data. Stefan Schulz assisted with the chemical analysis. Gabriela Montejo‐Kovacevich created the intraspecific genetic distance matrices. Richard Merrill, Owen McMillan, Kelsey Byers, and Chris Jiggins contributed to the project design. All authors contributed to manuscript revisions.

### Open Research Badge

This article has earned an Open Data Badge for making publicly available the digitally‐shareable data necessary to reproduce the reported results. The data is available at https://osf.io/28yfk/?view_only=c1f7e7a925e74dee84fd2229cbf3f511

## Supporting information

Table S1‐S2

## Data Availability

Sequencing data are available from ENA under accession number PRJEB35570. Other data supporting the findings of this study including GCMS chromatograms and R scripts used for analysis are available from Open Science Framework (OSF) at https://osf.io/28yfk/ (https://doi.org/10.17605/OSF.IO/28YFK).

## APPENDIX 1

This section contains sample numbers for both chemical and genetic analyses.

**Table A1:** Number of androconial and genital samples collected for each locality

Locality	Species	No. of androconial samples	No. of genital samples
Panama	*H. cydno*	12	12
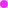
Panama	*H. erato*	7	9
Panama	*H. melpomene*	9	10
Panama	*H. sapho*	12	11
Colombia (highlands)	*H. erato*	10	9
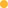
Colombia (highlands)	*H. melpomene*	13	14
Colombia (highlands)	*H. timareta*	7	8
Colombia (lowlands)	*H. erato*	12	13
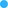
Colombia (lowlands)	*H. melpomene*	7	8
Ecuador (east)	*H. elevatus*	2	2
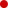
Ecuador (east)	*H. erato*	10	11
Ecuador (east)	*H. melpomene*	11	13
Ecuador (east)	*H. timareta*	2	3
Ecuador (west)	*H. cydno*	12	12
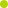
Ecuador (west)	*H. eleuchia*	9	8
Ecuador (west)	*H. erato*	12	13
Ecuador (west)	*H. melpomene*	11	12
Ecuador (west)	*H. sapho*	2	2
Peru (central)	*H. erato*	7	6
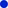
Peru (central)	*H. melpomene*	3	4
Peru (south)	*H. erato*	9	9
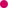
Peru (south)	*H. melpomene*	3	3
Brazil (Coastal Para)	*H. melpomene*	8	8
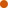
Brazil (Mato Grosso)	*H. erato*	12	12
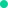
Brazil (Mato Grosso)	*H. melpomene*	10	15
Brazil (North Para)	*H. erato*	5	6
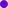
Brazil (North Para)	*H. melpomene*	13	15
Brazil (South Para)	*H. elevatus*	2	2
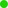
Brazil (South Para)	*H. erato*	4	5
Brazil (South Para)	*H. melpomene*	3	6
Brazil (Rondonia)	*H. elevatus*	3	3
Brazil (Rondonia)	*H. erato*	10	11

**Table A2:** Genome samples of *H. erato* and *H. melpomene* races including number of androconial (A) and genital (G) samples included in analysis for each race

Locality	Taxon name	A	G	ID	Lat.	Lon.	Accession
Colombia (highlands)	*H. m. bellula*	13	14	CAM040049*	1.217	−76.683	SAMEA6447026
Colombia (lowlands)	*H. m. malleti*	7	8	CS002311	1.814	−75.669	SAMEA3723397
Ecuador (east)	*H. m. malleti*	11	13	CAM016540	−1.061	−77.668	SAMEA2240083
Ecuador (west)	*H. m. cythera*	11	12	14N015*	0.185	−78.853	SAMEA6447028
Brazil (Mato Grosso)	*H. m. burchelli*	3	5	SR281*	−13.814	−56.404	SAMEA6447027
Brazil (Coastal Para)	*H. m. intersectus*	1	1	KK291*	−1.070	−46.745	SAMEA6493175
Brazil (South Para)	*H. m. madeira*	2	5	SR391*	−4.066	−54.847	SAMEA6447029
Brazil (North Para)	*H. m. melpomene*	13	15	SR178*	−1.937	−54.626	SAMEA6447030
Brazil (Mato Grosso)	*H. m. penelope*	4	7	SR358*	−13.691	−57.706	SAMEA6447031
Brazil (Coastal Para)	*H. m. thelxiope*	7	7	KK288*	−1.070	−46.745	SAMEA6493176
Peru (south)	*H. m. schunkei*	3	3	KK544*	−13.204	−70.768	SAMEA6447032
Peru (central)	*H. m. xenoclea*	3	4	KK309*	−11.0354	−75.407	SAMEA6447033
Panama	*H. m. rosina*	9	10	CAM001841	9.076	−79.659	SAMEA104585083
Colombia (highlands)	*H. e. dignus*	10	9	CAM040113*	1.214	−76.690	SAMEA6447018
Colombia (lowlands)	*H. e. lativitta*	12	13	CAM040160*	0.956	−76.409	SAMEA6447021
Ecuador (west)	*H. e. cyrbia*	12	13	CAM040545*	0.151	−78.770	SAMEA6447017
Ecuador (east)	*H. e. lativitta*	10	11	CAM041030*	−1.059	−77.702	SAMEA6447022
Brazil (South Para)	*H. e. amazona*	4	5	SR122*	−4.066	−54.847	SAMEA6493174
Brazil (Mato Grosso)	*H. e. phyllis*	10	8	SR230*	−10.891	−55.440	SAMEA6447024
Brazil (Rondonia)	*H. e. venustus*	9	10	SR314*	−12.806	−60.297	SAMEA6447025
Peru (south)	*H. e. amphitrite*	9	9	KK464*	−12.955	−72.656	SAMEA6447016
Peru (central)	*H. e. emma*	1	1	KK402*	−10.298	−74.935	SAMEA6447019
Peru (central)	*H. e. microclea*	4	4	KK338*	−11.055	−75.419	SAMEA6447023
Panama	*H. e. demophoon*	7	9	Pet_ED3	−9.129	79.715	SAMN05224182

Note

All samples were males. Newly sequenced individuals are denoted with a star next to their ID. More information on individuals can be found on the public database https://heliconius.ecdb.io/.

## APPENDIX 2

This section contains model selection tables for both PERMANOVA and ManyGLM analyses.

**Table A3:** Model selection table for PERMANOVA models based on AIC scores

Model	Residual sum of squares	DF	AIC
Interspecific androconia			
Chemical profile ~ Species + Region + (Region/Locality) + Species * Region + Species * (Region/Locality)	26.436	31	889.2356
**Chemical profile ~ Species + Region + (Region/Locality) + Species * Region**	28.185	23	889.3774
Chemical profile ~ Species + Region + (Region/Locality)	31.760	17	907.4649
Chemical profile ~ Species + Region	34.377	9	911.4244
Chemical profile ~ Species	38.07	6	931.1351
Interspecific genitals			
Chemical profile ~ Species + Region + (Region/Locality) + Species * Region + Species * (Region/Locality)	31.887	31	1,016.104
**Chemical profile ~ Species + Region + (Region/Locality) + Species * Region**	**33.748**	**23**	**1,015.703**
Chemical profile ~ Species + Region + (Region/Locality)	39.008	17	1,043.535
Chemical profile ~ Species + Region	42.151	9	1,048.847
Chemical profile ~ Species	47.165	6	1,073.751
*H. erato* androconia			
**Chemical profile ~ Region + (Region/Locality)**	8.79	9	211.2867
Chemical profile ~ Region	10.3793	3	213.9033
*H. erato* genitals			
**Chemical profile ~ Region + (Region/Locality)**	9.1105	9	223.2669
Chemical profile ~ Region	10.9982	3	228.5917
*H. melpomene* androconia			
Chemical profile ~ Region + (Region/Locality)	9.8523	11	223.0307
**Chemical profile ~ Region**	11.7698	3	222.502
*H. melpomene* genitals			
**Chemical profile ~ Region + (Region/Locality)**	12.3471	10	283.3958
Chemical profile ~ Region	14.5178	3	286.2391

Note

If two models were within two AIC points of each other, we chose the simpler model as the most parsimonious. Best fit models are highlighted in bold.

**Table A4:** Analysis of deviance model selection table for multivariate generalized linear models based on likelihood ratio tests

Model	ΔDeviance	Residual DF		*p*‐value
Interspecific androconia				
**Chemical profile ~ Species + Region + (Region/Locality) + Species * Region + Species * (Region/Locality)**		220		
Chemical profile ~ Species + Region + (Region/Locality) + Species * Region	−740.7	228		.001
Interspecific genitals				
Chemical profile ~ Species + Region + (Region/Locality) + Species * Region + Species * (Region/Locality)		243		
Chemical profile ~ Species + Region + (Region/Locality) + Species * Region	−1854	251		.063
**Chemical profile ~ Species + Region + (Region/Locality)**	−2787	257		.134
Chemical profile ~ Species + Region	−6481	265		.026
*H. erato* androconia				
**Chemical profile ~ Region + (Region/Locality)**		78		
Chemical profile ~ Region	−1091	84		.001
*H. erato* genitals				
**Chemical profile ~ Region + (Region/Locality)**		82		
Chemical profile ~ Region	−2721	88		.001
*H. melpomene* androconia				
**Chemical profile ~ Region + (Region/Locality)**		75		
Chemical profile ~ Region	−1582	83		.001
*H. melpomene* genitals				
**Chemical profile ~ Region + (Region/Locality)**		93		
Chemical profile ~ Region	−1777	100	.001	

Note

Each model is compared to the model above it. Best fit models are highlighted in bold.

## APPENDIX 3

This section contains tables showing results from pairwise PERMANOVA comparisons, intraspecific indicator analyses, and model results tables for all ManyGLM analyses.

**Table A5:** Pairwise PERMANOVA *p*‐values of between species androconial comparisons using Bonferroni correction

	*H. cydno*	*H. eleuchia*	*H. elevatus*	*H. erato*	*H. melpomene*	*H. sapho*
*H. eleuchia*	.021					
*H. elevatus*	.042	.021				
*H. erato*	.021	.021	.021			
*H. melpomene*	.021	.021	.021	.021		
*H. sapho*	.021	.021	.021	.021	.021	
*H. timareta*	.021	.021	.021	.021	.021	.021

Note

All values were significant at .05 significance level.

**Table A6:** Pairwise PERMANOVA *p*‐values of between species genital comparisons using Bonferroni correction

	*H. cydno*	*H. eleuchia*	*H. elevatus*	*H. erato*	*H. melpomene*	*H. sapho*
*H. eleuchia*	**.021**					
*H. elevatus*	**.021**	**.021**				
*H. erato*	**.021**	**.021**	**.021**			
*H. melpomene*	**.021**	**.021**	.273	**.021**		
*H. sapho*	**.021**	**.042**	**.021**	**.021**	**.021**	
*H. timareta*	**.021**	**.021**	**.021**	**.021**	**.021**	**.021**

Note

Significant results are highlighted in bold.

**Table A7:** Summary of androconial chemical bouquet analysis of all species using the ManyGLM approach including all significant explanatory variables

Parameter	Residual DF	DF	Deviance	*p*‐value	Compounds
Species	245	6	10,944	.001	Geranylgeranylacetone*, syringaldehyde, methyloctadecanal (RI = 2076), icosanal, octadecanal*, (*Z*)‐11‐icosenal, henicosane, methyloctadecanal (RI = 2064), unknown RI = 1,396, 1‐hexadecanol
Region	242	3	3,717	.001	Henicosane, tricosane, unknown ester RI = 1,188, unknown RI = 2,133, tetracosane, napthalene^a^, unknown RI = 1,366, unknown RI = 2,277, pentacosane
(Region/Locality)	231	11	2,826	.001	Hexadecadien−15‐olide, unknown RI = 1915, unknown hydrocarbon RI = 1962, 1‐hexadecanol, napthalene^a^, nonanal, (*Z*)‐13‐docosenal, methyl 4‐hydroxy‐3‐methoxybenzoate, (*Z*)‐β‐ocimene, unknown RI = 1,184
Species * Region	222	9	896	.005	Napthalene, methyl salicylate, henicosane, 1‐octadecanol, mellein, dihydroactinidiolide, 1‐hexadecanol, octadecanal, (*Z*)‐13‐docosen‐1‐ol, tricosane
Species * (Region/Locality)	220	21	741	.001	1‐Hexadecanol, pentacosane, 1‐octadecanol, methyl salicylate, henicosane

Note

The ten compounds that contribute the most to the deviance explained by a variable are listed for each variable in descending order of contribution. Compounds highlighted with * were also identified by an indicator analysis.

^a^ Naphthalene is a known flower volatile, but can also be introduced by contamination. Our blank samples never contained naphthalene, indicating the butterfly origin in our study.

**Table A8:** Summary of genital chemical bouquet analysis of all species using the ManyGLM approach including all significant explanatory variables

Parameter	Residual DF	DF	Deviance	*p*‐value	Compounds
Species	268	6	27,587	.001	Unknown terpene ester RI = 2,494*, unknown terpene ester RI = 2,139, henicosane, unknown pentyl ester RI = 2033, unknown terpene ester 2,435, unknown aromatic RI = 1,299, unknown terpene RI = 2,755, benzyl cyanide, unknown sesterterpene hydrocarbon RI = 2,370, (*E*)‐β‐ocimene*
Region	265	3	8,965	.001	Unknown RI = 2,840, 7,8‐dihydro‐β‐ionone, benzyl cyanide, unknown aromatic ester RI = 2,511, 2‐phenylethyl dodecanoate, unknown triterpene RI = 2,891, unknown aromatic ester RI = 2,718, hexadecane, unknown RI = 1,076, 3‐undecanone
(Region/Locality)	257	11	6,431	.016	(*Z*)‐β‐Ocimene, unknown RI = 1915, unknown hydrocarbon RI = 1,750, 18‐octadecanolide, henicosene (2068), 19‐methylicosyl acetate, napthalene, hexadecanoic acid, 3‐undecanone, unknown terpene ester RI = 2,310

Note

The ten compounds that contribute the most to the deviance explained by a variable are listed for each variable. Compounds highlighted with * were also identified by an indicator analysis.

**Table A9:** Androconial and genital compounds that are the best indicators of different geographic groups of *H. erato*

*Wings*	A: specificity	B: coverage	sqrtIV
Amazon			
Napthalene	0.787	0.870	0.827
East Andes			
1‐Hexadecanol & mellein	0.802	0.957	0.876
West Andes			
Unknown RI = 1704	0.950	0.833	0.890
Panama			
Benzylacetate	1	1	1
Unknown ester RI = 1,188	1	1	1
*Genitals*			
Amazon			
Napthalene	0.851	0.957	0.902
East Andes			
Unknown triterpene RI = 2,891	0.846	0.978	0.910
West			
Unknown RI = 1833	1	1	1
Unknown RI = 1970	1	1	1
2‐Phenylethyl decanoate	1	1	1
Unknown terpene ester RI = 2,120	1	1	1
2‐Phenylethyl dodecanoate	1	1	1
Unknown RI = 2,258	1	1	1
2‐Phenylethyl tetradecenoate	1	1	1
Unknown aromatic ester RI = 2,511	1	1	1
Unknown aromatic ester RI = 2,718	1	1	1
Unknown RI = 2,734	1	1	1
Panama			
Benzyl acetate	1	1	1
Unknown ester RI = 1,188	1	1	1
Pentyl/isopentyl 3‐methylbutyrate	1	1	1

Note

A is a measure of group specificity of the compounds, B is a measure of group coverage, and sqrtIV is the indicator value that considers both A and B and ranges from 0 (compound not present in any individuals of that species) to 1 (compound only present in that species, and present in all individuals).

**Table A10:** Androconial and genital compounds that are the best indicators of different geographic groups of *H. melpomene*

*Wings*	A: specificity	B: coverage	sqrtIV
Amazon			
Alkene or alcohol (RI = 2,127) & henicosane	0.871	0.966	0.917
East Andes			
(*Z*)‐13‐Docosenal & henicosane	0.962	0.895	0.928
West Andes			
Unknown RI = 1766	1	0.727	0.852
Panama			
Nonanoic acid	0.816	1	0.903
*Genitals*			
Amazon			
14‐Tetradecanolide	0.915	0.975	0.945
East Andes			
7,8‐Dihydro‐β‐ionone	1	0.881	0.939
West			
Hexyl octadecanoate (RI = 2,621)	0.826	1	0.909
Panama			
2‐s‐Butyl‐3‐methoxypyrazine	0.495	1	0.704

Note

A is a measure of group specificity of the compounds, B is a measure of group coverage, and sqrtIV is the indicator value that considers both A and B and ranges from 0 (compound not present in any individuals of that species) to 1 (compound only present in that species, and present in all individuals).

**Table A11:** Summary of androconial chemical bouquet analysis of *H. erato* using the ManyGLM approach including all significant explanatory variables

Parameter	Residual DF	DF	Deviance	p‐val	Compounds
Region	84	3	1,275	0.001	Pentacosane, unknown ester RI = 1,188*, benzyl acetate*, unknown macrolide RI = 1714, tetracosane, unknown RI = 1,366, pentyl/isopentyl 3‐ethylbutyrate RI = 1,145, mellein*, hexadecadien−15‐olide, heptadecene,
(Region/Locality)	78	9	1,091	0.001	Hexadecadien−15‐olide, napthalene*, unknown RI = 1915, unknown hydrocarbon RI = 1962, tetracosane, unknown RI = 1,184, unknown macrolide RI = 1714, pentacosane, unknown RI = 1,444, unknown RI = 1,424

Note

The ten compounds that contribute the most to the deviance explained by a variable are listed for each variable. Compounds highlighted with * were also identified by an indicator analysis.

**Table A12:** Summary of genital chemical bouquet analysis of *H. erato* using the ManyGLM approach including all significant explanatory variables

Parameter	Residual DF	DF	Deviance	p‐val	Compounds
Region	88	3	6,142	0.001	3‐Undecanone, unknown sesterterpene RI = 2,636*, unknown RI = 2,840, unknown triterpene RI = 2,891*, unknown aromatic ester RI = 2,511*, 2‐phenylethyl dodecanoate*, unknown RI = 2,451, unknown terpene ester RI = 2,435, unknown aromatic ester RI = 2,718*, benzyl cyanide,
(Region/Locality)	82	9	2,721	0.001	Napthalene*, hexadecanoic acid, unknown diterpene RI = 2,205, unknown ester hexanoate RI = 1565, 18‐octadecanolide, unknown RI = 2,279, unknown macrolide RI = 1714, unknown RI = 1,424, unknown amide RI = 2,157, icosanal

Note

The ten compounds that contribute the most to the deviance explained by a variable are listed for each variable. Compounds highlighted with * were also identified by an indicator analysis.

**Table A13:** Summary of androconial chemical bouquet analysis of *H. melpomene* using the ManyGLM approach including all significant explanatory variables

Parameter	Residual DF	DF	Deviance	p‐val	Compounds
Region	83	3	1,848	0.001	Henicosane*, tricosane, methyl 3,4‐dimethoxybenzoate, homovanillyl alcohol, (*Z*)−13‐docosen−1‐ol*, 11‐icosenol, icosenol, napthalene, unknown aromatic RI = 1738, unknown alkene or alcohol RI = 2,127*
(Region/Locality)	75	11	1,582	0.001	Unknown RI = 2,133, nonanal, 1‐octadecanol, (*Z*)−13‐docosenal, (*Z*)−9‐octadecenal, Unknown RI = 1915, (*Z*)−16‐methyl−9‐octadecenol, unknown RI = 2,112, unknown RI = 1638, tricosene RI = 2072

Note

The ten compounds that contribute the most to the deviance explained by a variable are listed for each variable.

**Table A14:** Summary of genital chemical bouquet analysis of *H. melpomene* using the ManyGLM approach including all significant explanatory variables

Parameter	Residual DF	DF	Deviance	p‐val	Compounds
Region	100	3	2,281	0.001	7,8‐Dihydro‐β‐ionone*, 12‐dodecanolide, 2‐*s*‐butyl−3‐methoxypyrazine, napthalene, unknown RI = 1704, 2‐methoxy−3‐isobutylpyrazine*, (*Z*)‐β‐ocimene, unknown RI = 1607, 11‐dodecanolide, (*E*)‐β‐ocimene
(Region/Locality)	93	10	1,777	0.001	12‐Dodecanolide, unknown hydrocarbon RI = 1,750, unknown RI = 1915, benzyl salicylate, hexenyl octadecatrienoate & (Z)−3‐hexenyl octadecenoate, 11‐methylpentacosane, unknown RI = 2,891, 14‐tetradecanolide*, unknown sesquiterpene RI = 1902, 11‐dodecanolide

Note

The ten compounds that contribute the most to the deviance explained by a variable are listed for each variable. Compounds highlighted with * were also identified by an indicator analysis.

## APPENDIX 4

We compared within and between species and locality Bray–Curtis distances. We focused on *H. erato* and *H. melpomene*, as these species were collected in the most localities. We calculated a Bray–Curtis distance matrix and then used the function “dist_groups” from the package *usedist* to calculate distances between individuals of different groups (Bittinger, 2017). We add statistical comparisons to the violin plots using the function “stat_compare_means” from the package *ggpubr* (Kassambara, 2019). For both androconia and genitals, the mean chemical distance between individuals is greater between species (androconia, 0.971; genitals, 0.915) than within species (androconia, 0.554; genitals, 0.573). The mean chemical distance between individuals is also greater between localities (androconia, 0.564; genitals, 0.584) than within localities (androconia, 0.457; genitals, 0.466). However, the magnitude of this difference is much smaller, confirming the PERMANOVA and ManyGLM analyses that most variation is explained by species and not geographic location.

## APPENDIX 5

Here, we rerun statistical analyses excluding unknown compounds or poorly sampled populations.

### Does removing unidentified compounds or poorly sampled populations affect models of interspecific variation in chemical profiles?

We repeated the interspecific analysis without unidentified compounds. When repeated without unidentified compounds, species still significantly differ in their androconial bouquet, with species identity accounting for 60% of the overall variation in chemical profiles (PERMANOVA, Species, *F*_6,251_ = 77.33, *p* < .001). A further 4% of variation can be explained by region (Amazon/Eastern Andes/Western Andes/Panama), and 3% by locality nested within region (PERMANOVA, Region, *F*_3,251_ = 9.96, *p* < .001 (Region/Locality), *F*_8,251_ = 2.49, *p* < .001). Finally, 4% of variation is explained by an interaction between species and region (PERMANOVA, Species * Region *F*_6,251_ = 4.77, *p* < .001). Results were also similar for genital bouquets, with species identity still explaining the highest amount of variation, accounting for 44% of the variation in chemical profiles (PERMANOVA, Species, *F*_6,274_ = 45.44, *p* < .001). A further 6% of variation can be explained by region (Amazon/Eastern Andes/Western Andes/Panama), and 4% by locality nested within region (PERMANOVA, Region, *F*_3,274_ = 12.74, *p* < .001 (Region/Locality), *F*_8,274_ = 2.85, *p* < .001). Finally, 6% of variation is explained by an interaction between species and region (PERMANOVA, Species * Region *F*_6,274_ = 6.32, *p* < .001).

We also repeated the interspecific analyses removing populations with fewer than 5 individuals. Again, similar to removing unidentified compounds, and the full dataset, species identity accounts for 58% of overall variation in chemical profiles, with a further 5% explained by region, 3% by locality nested within region, and 4% by an interaction between species and region ( PERMANOVA, Species, *F*_5,227_ = 78.26, *p* < .001; Region, *F*_3,227_ = 10.19, *p* < .001 (Region/Locality), *F*_7,227_ = 2.98, *p* < .001; Species * Region *F*_4,227_ = 6.92, *p* < .001). Results were also consistent for genital bouquets, with species identity still explaining the highest amount of variation, accounting for 51% of the variation in chemical profiles, with a further 5% explained by region, 4% by locality nested within region, and 5% by an interaction between species and region (PERMANOVA, Species, *F*_5,255_ = 69.49, *p* < .001; Region, *F*_3,255_ = 12.37, *p* < .001 (Region/Locality), *F*_8,255_ = 3.05, *p* < .001; Species * Region *F*_4,255_ = 9.13, *p* < .001).

### Does removing unidentified compounds or poorly sampled populations affect correlations between divergence in chemical profile with genetic and geographic distance?

Correlations with genetic and geographic distances were also consistent with results including all compounds. When controlling for geographic distance, genetic divergence is strongly correlated with both androconial and genital chemical divergence (Mantel test, androconia, *r* = .7897, *p* = .001; genitals, *r* = .5203, *p* = .001). When controlling for genetic distance, geographic distance is significantly but weakly correlated with chemical divergence (Mantel test, androconia, *r* = .06739, *p* = .002; genitals, *r* = .059, *p* = .003).

Removing populations with fewer than 5 individuals also gave consistent results. When controlling for geographic distance, genetic and chemical divergence remain strongly correlated (Mantel test, androconia, *r* = .7978, *p* = .001; genitals, *r* = .71, *p* = .001). Again, when controlling for genetic distance, geographic distance is significantly but weakly correlated with androconial and genital chemical divergence (partial Mantel test, androconia, *r* = .082, *p* = .001; genitals, *r* = .0439, *p* = .006).

### Does removing unidentified compounds affect models of intraspecific variation in chemical profiles?

Individuals of *H. erato* still strongly group by region when unidentified compounds are removed from the analysis, with 27% of variation in androconial profiles being explained by region and 11% by locality nested within region (PERMANOVA, Region *F*_3,87_ = 11.49, *p* < .001, Locality *F*_6,87_ = 2.30, *p* < .001). Again, this is similar for *H. erato* genital compounds. Region explains 35% of variation, and 8% is explained by locality nested within region (PERMANOVA, Region *F*_3,91_ = 16.76, *p* < .001, Locality *F*_6,91_ = 1.98, *p* < .01).

Again, we found consistent results without unidentified compounds for *H. melpomene.* The same amount of variation was explained in models with and without unidentified compounds included, with region explaining 18% of variation in androconial compounds (PERMANOVA, Region *F*_3,86_ = 6.05, *p* < .01). For *H. melpomene* genital compounds, 20% of variation is explained by region, 12% by locality nested within region, as in models with all compounds included (PERMANOVA, Region *F*_3,103_ = 9.05, *p* < .001, Locality *F*_7,103_ = 2.34, *p* < .001).

### Does removing unidentified compounds affect correlations between intraspecific chemical divergence with genetic and geographic distance?

Results were again very similar without unidentified compounds included in the analysis. In *H. erato,* both androconial and genital chemical distance are positively correlated with genetic distance, even when accounting for geographic distance (partial Mantel test, androconia, *R* = .148, *p* = .001; genitals, *R* = .280, *p* = .001). When unidentified compounds are removed, we still find a weak positive correlation between geographic distance and androconial, but not genital distance, accounting for genetic distance (partial Mantel test, androconia, *R* = .155, *p* = .002; genitals, *R* = −.0171, *p* = .656).

For *H. melpomene*, genital bouquet divergence, but not androconial bouquet divergence, is correlated with genetic distance when accounting for geography (partial Mantel test, androconia, *R* = .02602, *p* = .169, genitals, *R* = .112, *p* = .001). When we first consider genetic distance, geographic distance is not positively correlated with genital chemical distance; however, it is positively correlated with androconial chemical distance (partial Mantel test, androconia, *R* = .1729, *p* = .002; genitals, *R* = .003, *p* = .439). These results are consistent with tests including all compounds.
