# Supplementary material for: Species specificity and intraspecific variation in the chemical profiles of Heliconius butterflies across a large geographic range
Source: Ecol Evol. 2020 Apr 3;10(9):3895–918. doi: 10.1002/ece3.6079 (PMC7244815; doi:10.1002/ece3.6079)
Supplement: Supplementary file 1 — Table S1‐S2 [file ECE3-10-3895-s001.docx]

**Supplementary information**

**Table S1: Androconial compounds identified in at least half of all individuals from any population. The Kovats gas chromatographic retention index (RI) is reported for each compound. Mean amounts (ng) in *H. cydno* (CYD), *H. eleuchia* (ELEU), *H. elevatus* (ELEV), *H. erato* (ERAT), *H. melpomene* (MEL), *H. sapho* (SAPH), *H. timareta* (TIM).**

| **Compound** | **RI** | **CYD** | **ELEU** | **ELEV** | **ERAT** | **MEL** | **SAPH** | **TIM** |
| --- | --- | --- | --- | --- | --- | --- | --- | --- |
| Unknown | 1018 | 0.00 | 0.00 | 0.00 | 0.63 | 0.37 | 0.00 | 0.00 |
| (Z)-β-Ocimene | 1030 | 0.00 | 0.00 | 123.97 | 0.24 | 40.92 | 0.00 | 0.00 |
| Phenylacetaldehyde | 1036 | 1.03 | 0.26 | 0.00 | 0.07 | 0.02 | 0.00 | 1.30 |
| Acetophenone | 1065 | 0.00 | 0.00 | 0.00 | 0.06 | 0.00 | 0.00 | 0.00 |
| cis-Linalool oxide | 1082 | 0.88 | 0.00 | 3.71 | 0.00 | 0.00 | 0.00 | 0.00 |
| o-Guaiacol | 1090 | 0.08 | 0.00 | 0.00 | 0.00 | 0.39 | 0.03 | 0.22 |
| Nonanal | 1105 | 3.45 | 0.75 | 3.91 | 2.87 | 21.05 | 1.02 | 15.41 |
| 3E,5E-2,6-Dimethyl-1,3,5,7-octatraen | 1120 | 0.00 | 0.00 | 0.59 | 0.00 | 0.23 | 0.00 | 0.00 |
| Phenylacetonitrile | 1139 | 6.88 | 0.00 | 0.86 | 0.08 | 0.00 | 0.00 | 8.49 |
| Unknown | 1145 | 0.00 | 0.00 | 0.00 | 0.89 | 0.00 | 0.00 | 0.00 |
| Benzyl acetate | 1165 | 0.00 | 0.00 | 0.00 | 0.61 | 0.19 | 0.10 | 0.00 |
| Napthalene | 1175 | 6.04 | 1.20 | 8.29 | 3.45 | 4.66 | 1.87 | 0.38 |
| Unknown | 1184 | 0.00 | 0.00 | 0.00 | 3.19 | 0.00 | 0.00 | 0.00 |
| Unknown | 1188 | 0.04 | 0.00 | 0.00 | 8.80 | 1.17 | 0.32 | 0.00 |
| Methyl Salicylate | 1189 | 9.72 | 2.99 | 12.43 | 2.62 | 2.82 | 1.58 | 0.74 |
| Decanal | 1198 | 0.12 | 0.00 | 0.00 | 0.00 | 0.84 | 0.16 | 0.00 |
| 4-Vinylphenol | 1215 | 0.00 | 13.98 | 0.00 | 0.00 | 0.00 | 2.71 | 0.00 |
| Benzothiazole | 1215 | 0.00 | 0.00 | 0.00 | 0.01 | 0.13 | 0.00 | 0.00 |
| Unknown | 1236 | 0.00 | 0.00 | 0.00 | 0.05 | 0.03 | 0.00 | 7.28 |
| Unknown | 1268 | 1.47 | 0.00 | 0.00 | 0.01 | 0.54 | 0.00 | 0.00 |
| Nonanoic acid | 1269 | 0.00 | 0.00 | 0.24 | 0.02 | 1.24 | 0.00 | 0.00 |
| 3-Undecanone | 1275 | 0.00 | 0.00 | 0.00 | 13.79 | 0.00 | 0.00 | 0.08 |
| 1-Tridecene | 1290 | 0.00 | 0.00 | 0.00 | 4.26 | 0.00 | 0.00 | 0.00 |
| Indole | 1295 | 0.00 | 0.00 | 0.04 | 0.00 | 0.19 | 0.00 | 0.00 |
| Unknown aromatic | 1299 | 0.00 | 0.00 | 0.72 | 0.43 | 0.00 | 0.00 | 0.15 |
| Unknown | 1353 | 0.00 | 0.00 | 0.00 | 0.06 | 0.18 | 0.00 | 1.52 |
| Glycerol ester | 1356 | 0.20 | 0.00 | 0.00 | 0.87 | 0.53 | 0.47 | 102.62 |
| Unknown | 1366 | 0.04 | 0.00 | 0.00 | 0.22 | 0.14 | 0.00 | 0.68 |
| 1,3-Dimethyluracil | 1371 | 0.00 | 0.00 | 0.37 | 0.40 | 0.05 | 0.03 | 0.00 |
| Unknown | 1371 | 0.13 | 0.00 | 0.00 | 0.06 | 0.02 | 0.00 | 4.47 |
| Unknown alkene | 1382 | 0.00 | 0.00 | 0.00 | 0.97 | 0.00 | 0.00 | 0.00 |
| Unknown ester | 1392 | 0.00 | 8.58 | 0.00 | 0.00 | 0.00 | 7.27 | 0.00 |
| Butanoic acid ester | 1394 | 0.00 | 0.16 | 0.00 | 1.16 | 0.00 | 0.60 | 0.00 |
| Unknown | 1396 | 0.06 | 0.00 | 0.00 | 10.72 | 0.07 | 0.00 | 0.00 |
| Unknown | 1424 | 0.00 | 0.00 | 0.00 | 1.10 | 0.00 | 0.00 | 0.00 |
| 2-Methoxy-4-propylphenol | 1436 | 0.00 | 0.00 | 24.40 | 0.00 | 0.02 | 0.00 | 0.00 |
| Unknown | 1444 | 0.44 | 0.00 | 0.00 | 1.79 | 0.04 | 0.00 | 0.88 |
| Unknown | 1445 | 0.10 | 0.00 | 0.00 | 0.00 | 0.68 | 0.69 | 0.08 |
| Unknown | 1483 | 0.00 | 0.00 | 0.00 | 0.00 | 0.00 | 0.00 | 1.34 |
| 5-Decanolide | 1493 | 0.00 | 0.00 | 0.00 | 0.00 | 0.00 | 0.00 | 22.31 |
| Methyl 4-hydroxy- methoxybenzoate | 1517 | 0.00 | 0.00 | 0.00 | 1.35 | 0.12 | 66.11 | 0.00 |
| Unknown aromatic | 1519 | 0.00 | 0.00 | 0.00 | 0.00 | 2.87 | 0.00 | 0.00 |
| Unknown aromatic | 1519 | 0.07 | 0.00 | 0.00 | 0.00 | 2.98 | 0.00 | 0.00 |
| d-Cadinene | 1522 | 0.00 | 0.00 | 0.00 | 1.81 | 0.06 | 0.00 | 0.00 |
| Homovanillyl alcohol | 1523 | 0.04 | 0.00 | 695.61 | 0.74 | 4.02 | 0.00 | 3.23 |
| Dihydroactinidiolide | 1532 | 8.57 | 0.48 | 0.00 | 44.91 | 22.69 | 5.88 | 28.55 |
| Calacorene | 1540 | 0.00 | 0.00 | 0.21 | 0.04 | 0.04 | 0.00 | 0.00 |
| Mellein | 1540 | 0.00 | 44.75 | 89.60 | 179.38 | 27.21 | 14.19 | 0.00 |
| Unknown aromatic | 1563 | 0.73 | 0.00 | 0.31 | 0.00 | 0.20 | 0.08 | 0.00 |
| Unknown | 1571 | 0.00 | 0.00 | 0.00 | 0.00 | 0.33 | 0.00 | 0.00 |
| Unknown alkene | 1581 | 0.00 | 0.00 | 0.00 | 0.68 | 0.00 | 0.00 | 0.00 |
| Methyl 3,4- dimethoxybenzoate | 1582 | 0.00 | 0.00 | 0.00 | 0.00 | 4.74 | 0.00 | 0.00 |
| Unknown | 1638 | 0.00 | 0.00 | 0.00 | 0.01 | 1.18 | 0.00 | 2.40 |
| Unknown | 1639 | 0.00 | 0.00 | 1.18 | 0.76 | 0.42 | 0.15 | 1.83 |
| d-Cadinol | 1644 | 0.34 | 0.00 | 0.00 | 157.03 | 0.09 | 0.00 | 0.00 |
| Unknown | 1649 | 0.00 | 0.00 | 0.00 | 0.00 | 0.25 | 0.00 | 0.00 |
| Syringaldehyde | 1661 | 937.77 | 0.00 | 574.45 | 0.30 | 488.42 | 183.68 | 1043.19 |
| Heptadecene | 1679 | 0.00 | 0.00 | 0.46 | 0.57 | 0.23 | 0.00 | 0.00 |
| Unknown | 1704 | 0.00 | 0.00 | 0.00 | 2.30 | 0.00 | 0.00 | 0.00 |
| 3,5-Dimethoxy-4- hydroxybenzyl alcohol | 1707 | 4.93 | 0.00 | 1.91 | 0.01 | 14.08 | 9.31 | 52.82 |
| Unknown | 1714 | 0.00 | 0.00 | 0.00 | 3.97 | 0.00 | 0.00 | 0.00 |
| 1-(3,5-Dimethoxy-4-hydroxybenzyl) ethanone | 1735 | 0.79 | 0.00 | 1.59 | 0.00 | 1.42 | 0.00 | 0.54 |
| Unknown aromatic | 1738 | 0.00 | 0.00 | 0.00 | 0.00 | 0.85 | 0.00 | 0.00 |
| Unknown hydrocarbon | 1750 | 1.62 | 1.12 | 3.38 | 3.86 | 2.60 | 2.59 | 4.37 |
| 1*H*-Indol-3-ethanol | 1754 | 0.00 | 0.00 | 0.21 | 0.00 | 0.90 | 0.00 | 0.00 |
| Unknown aromatic | 1757 | 0.00 | 0.00 | 0.00 | 0.00 | 0.45 | 0.00 | 0.00 |
| Ethyl benzoate | 1762 | 0.42 | 0.00 | 1.69 | 0.90 | 0.45 | 1.38 | 0.73 |
| Benzyl benzoate | 1766 | 0.00 | 0.00 | 0.00 | 0.00 | 0.32 | 0.10 | 0.00 |
| Unknown | 1766 | 0.00 | 0.13 | 0.00 | 0.00 | 0.52 | 0.00 | 0.00 |
| Unknown | 1771 | 0.00 | 0.00 | 0.70 | 0.17 | 0.13 | 0.03 | 0.00 |
| Unknown | 1780 | 0.00 | 0.00 | 0.00 | 0.25 | 0.00 | 0.00 | 0.00 |
| Unknown aromatic | 1797 | 0.00 | 0.00 | 0.00 | 0.00 | 0.21 | 0.06 | 2.87 |
| Octadecane | 1800 | 0.02 | 0.00 | 2.94 | 1.06 | 0.80 | 0.03 | 0.00 |
| Hexadecadien-15-olide | 1807 | 0.00 | 0.00 | 0.00 | 54.93 | 0.00 | 0.00 | 0.00 |
| Unknown aromatic sulfur | 1807 | 0.65 | 0.00 | 0.20 | 0.01 | 2.05 | 0.00 | 0.10 |
| Hexadecanal | 1815 | 0.00 | 0.00 | 0.00 | 2.32 | 0.55 | 0.00 | 0.00 |
| Methyl 1*H*-indol-3-acetate | 1822 | 0.00 | 0.00 | 0.00 | 0.00 | 1.44 | 0.00 | 0.34 |
| Neophytadiene | 1836 | 0.00 | 399.37 | 0.00 | 0.00 | 0.00 | 24.48 | 0.00 |
| Unknown | 1841 | 0.13 | 0.00 | 0.00 | 0.04 | 0.00 | 0.00 | 2.93 |
| Hexahydrofarnesylacetone | 1843 | 0.00 | 813.34 | 0.00 | 0.00 | 0.00 | 177.49 | 0.00 |
| Methyl 1*H*-indol-3-carboxylate | 1853 | 0.00 | 0.00 | 5.54 | 0.03 | 1.49 | 0.00 | 1.56 |
| Neophytadiene | 1861 | 0.00 | 102.95 | 0.00 | 0.00 | 0.00 | 7.06 | 0.00 |
| Benzyl salicylate | 1870 | 0.23 | 20.44 | 5.48 | 0.18 | 4.92 | 51.30 | 0.00 |
| Unknown branched alcohol | 1873 | 0.00 | 0.00 | 0.00 | 0.00 | 1.29 | 0.00 | 0.00 |
| Nonadecene | 1877 | 0.00 | 0.00 | 0.51 | 0.52 | 0.21 | 0.00 | 0.00 |
| Neophytadiene | 1878 | 0.00 | 163.30 | 0.00 | 0.00 | 0.00 | 8.71 | 0.00 |
| 1-Hexadecanol | 1884 | 0.00 | 0.00 | 1.15 | 479.25 | 2.53 | 0.00 | 0.00 |
| Nonadecane | 1899 | 7.34 | 0.00 | 11.06 | 0.43 | 0.23 | 0.00 | 0.00 |
| Unknown | 1915 | 0.05 | 0.00 | 3.35 | 1.73 | 1.11 | 0.99 | 0.00 |
| Heptadecanal | 1918 | 0.00 | 1.61 | 0.00 | 0.00 | 3.70 | 0.00 | 0.00 |
| Hexadecanoic acid | 1960 | 1.28 | 0.49 | 52.75 | 0.81 | 0.76 | 0.00 | 0.00 |
| Unknown hydrocarbon | 1962 | 0.00 | 0.00 | 0.54 | 0.58 | 0.24 | 0.71 | 0.00 |
| Unknown | 1962 | 0.21 | 0.26 | 0.55 | 0.73 | 0.16 | 0.58 | 0.76 |
| Unknown | 1970 | 2.95 | 0.00 | 0.00 | 0.00 | 0.00 | 0.00 | 0.00 |
| Unknown | 1979 | 0.00 | 0.00 | 0.00 | 0.79 | 2.03 | 0.00 | 0.00 |
| (Z)-9-Octadecenal | 1990 | 0.51 | 0.00 | 0.00 | 0.00 | 143.53 | 0.00 | 0.00 |
| Hexadecylacetate | 1991 | 0.00 | 0.00 | 0.00 | 4.79 | 0.00 | 0.00 | 0.00 |
| Icosane | 2000 | 15.52 | 0.00 | 37.08 | 0.09 | 0.20 | 0.00 | 0.00 |
| Octadecanal | 2013 | 0.68 | 0.00 | 0.00 | 0.34 | 3703.41 | 12.65 | 5.88 |
| Isopropyl Palmitate | 2020 | 6.55 | 2.28 | 0.00 | 0.11 | 0.00 | 0.00 | 0.00 |
| Unknown | 2036 | 1.12 | 0.00 | 0.00 | 0.00 | 0.00 | 0.00 | 0.00 |
| 9-Octadecen-1-ol | 2042 | 0.00 | 0.00 | 0.00 | 0.00 | 38.95 | 0.00 | 0.00 |
| Unknown macrolide | 2045 | 0.00 | 0.00 | 0.00 | 1.17 | 0.00 | 0.00 | 0.00 |
| Unknown | 2047 | 1.13 | 0.00 | 0.00 | 0.00 | 0.00 | 0.00 | 0.00 |
| Nonadecanal (methyl, branched) | 2054 | 0.00 | 0.00 | 0.00 | 0.00 | 22.87 | 0.00 | 0.00 |
| Ethyl 4-hydroxy-3,5- dimethoxybenzoate | 2057 | 1.54 | 0.00 | 3.48 | 0.10 | 12.00 | 0.00 | 1.37 |
| Methyloctadecanal | 2064 | 0.00 | 0.00 | 0.00 | 0.00 | 45.62 | 0.00 | 0.00 |
| Henicosene | 2068 | 1.02 | 0.00 | 2.97 | 0.95 | 0.00 | 0.00 | 0.00 |
| Henicosene | 2072 | 0.55 | 0.00 | 4.00 | 1.65 | 0.00 | 0.00 | 0.00 |
| Methyloctadecanal | 2072 | 0.00 | 0.00 | 0.00 | 0.00 | 20.58 | 0.00 | 0.00 |
| Methyloctadecanal | 2077 | 0.00 | 0.00 | 0.00 | 0.00 | 152.20 | 0.00 | 0.00 |
| 1-Octadecanol | 2083 | 0.00 | 0.00 | 2.20 | 39.87 | 1189.47 | 4.59 | 0.42 |
| Henicosene | 2086 | 0.40 | 0.00 | 8.14 | 0.86 | 0.10 | 0.00 | 0.00 |
| (*Z*)-16-Methyl- | 2092 | 0.00 | 0.00 | 0.00 | 0.00 | 31.95 | 0.00 | 0.00 |
| 9-octadecenol |  |  |  |  |  |  |  |  |
| Henicosane | 2100 | 2543.56 | 0.39 | 4554.83 | 0.71 | 181.97 | 1.02 | 141.39 |
| Unknown | 2110 | 12.18 | 0.00 | 0.00 | 0.00 | 0.00 | 0.00 | 0.00 |
| (*E*)-Phytol | 2112 | 0.00 | 1071.10 | 0.00 | 0.00 | 0.00 | 680.94 | 0.00 |
| Unknown | 2112 | 0.00 | 0.00 | 0.00 | 0.00 | 15.88 | 0.65 | 0.00 |
| Unknown alkene/alcohol | 2127 | 0.00 | 0.00 | 0.00 | 0.00 | 68.88 | 0.00 | 0.00 |
| Unknown aromatic | 2130 | 2.83 | 0.00 | 0.00 | 0.00 | 0.00 | 0.00 | 0.00 |
| Unknown | 2133 | 0.00 | 0.00 | 0.00 | 0.00 | 97.03 | 0.00 | 0.00 |
| Unknown amide | 2134 | 0.12 | 0.00 | 0.74 | 0.13 | 0.10 | 0.00 | 0.00 |
| Methyloctadecan-1-ol | 2138 | 0.00 | 0.00 | 0.00 | 0.00 | 32.53 | 0.00 | 0.00 |
| Phytenal | 2141 | 0.00 | 5.17 | 0.00 | 0.00 | 0.00 | 4.56 | 0.00 |
| Unknown amide | 2157 | 0.12 | 0.21 | 1.28 | 0.32 | 0.39 | 0.00 | 0.00 |
| Unknown | 2160 | 0.00 | 0.00 | 0.46 | 0.14 | 0.17 | 0.00 | 0.00 |
| Ethyl oleate | 2160 | 0.00 | 0.00 | 0.00 | 0.00 | 3.01 | 0.00 | 0.00 |
| Unknown | 2165 | 0.75 | 30.24 | 0.00 | 0.05 | 0.00 | 0.00 | 0.00 |
| Unknown | 2180 | 0.19 | 0.00 | 0.00 | 0.00 | 0.00 | 0.00 | 0.00 |
| Unknown | 2189 | 0.00 | 0.00 | 0.32 | 0.38 | 0.23 | 0.00 | 0.00 |
| (*Z*)-11-Icosenal | 2192 | 4.47 | 0.00 | 0.38 | 0.00 | 311.60 | 0.00 | 0.00 |
| Docosane | 2200 | 12.47 | 0.00 | 48.09 | 1.85 | 2.83 | 0.20 | 0.58 |
| Unknown | 2215 | 0.00 | 0.00 | 2.72 | 0.00 | 1.85 | 0.22 | 0.00 |
| Phytol | 2217 | 0.00 | 10.72 | 0.00 | 0.00 | 0.00 | 1.98 | 0.00 |
| Icosenol | 2255 | 20.19 | 0.00 | 7.95 | 0.00 | 174.52 | 0.11 | 6.54 |
| 17-Methyloctadecylacetate | 2271 | 0.00 | 0.00 | 0.00 | 0.00 | 2.71 | 0.00 | 0.00 |
| Tricosene | 2276 | 1.56 | 0.00 | 3.66 | 0.39 | 18.96 | 0.00 | 0.00 |
| Unknown | 2277 | 0.00 | 0.00 | 0.38 | 0.61 | 0.72 | 0.00 | 0.00 |
| Icosanal | 2283 | 0.00 | 0.00 | 0.00 | 0.01 | 118.07 | 6.57 | 0.00 |
| Tricosane | 2300 | 73.71 | 1.46 | 243.92 | 5.94 | 13.71 | 0.15 | 1.27 |
| Unknown | 2310 | 0.00 | 0.00 | 0.00 | 19.66 | 0.00 | 0.00 | 0.00 |
| 11-Icosenol | 2314 | 0.00 | 0.00 | 0.00 | 0.00 | 1.86 | 0.00 | 0.00 |
| Unknown fatty acid amide | 2325 | 0.98 | 0.89 | 1.20 | 1.04 | 0.57 | 1.35 | 0.70 |
| Unknown | 2346 | 0.00 | 0.19 | 0.14 | 0.12 | 0.24 | 0.30 | 0.00 |
| Unknown amide | 2347 | 4.61 | 0.00 | 60.92 | 28.34 | 29.68 | 0.00 | 0.00 |
| Unknown terpene | 2351 | 0.00 | 0.00 | 0.00 | 28.82 | 0.00 | 0.00 | 0.00 |
| Unknown | 2353 | 5.58 | 4.99 | 7.90 | 4.72 | 8.51 | 3.96 | 6.57 |
| Unknown | 2354 | 0.00 | 0.00 | 0.00 | 1.68 | 0.00 | 0.00 | 0.00 |
| Unknown amide | 2363 | 0.00 | 0.00 | 0.18 | 0.17 | 0.24 | 0.00 | 0.00 |
| Geranylgeranylacetone | 2382 | 0.00 | 0.00 | 0.00 | 304.07 | 0.00 | 0.00 | 0.00 |
| (*Z*)-13-Docosenal | 2396 | 0.39 | 0.00 | 0.00 | 0.00 | 87.58 | 0.00 | 0.00 |
| Tetracosane | 2400 | 0.30 | 0.32 | 13.32 | 12.99 | 5.22 | 0.00 | 7.02 |
| 19-Methylicosylacetate | 2422 | 0.00 | 37.61 | 0.00 | 3.20 | 4.25 | 2.37 | 0.00 |
| (*Z*)-13-Docosen-1-ol | 2464 | 0.00 | 0.00 | 0.00 | 0.88 | 18.48 | 1.68 | 0.00 |
| Unknown | 2490 | 0.00 | 0.00 | 0.58 | 0.39 | 0.03 | 0.00 | 0.00 |
| Pentacosane | 2500 | 4.39 | 16.62 | 44.57 | 30.48 | 13.06 | 4.49 | 12.00 |
| 11-Methylpentacosane | 2532 | 0.00 | 2.12 | 70.91 | 0.00 | 0.88 | 0.18 | 0.00 |

**Table S2 – Genital compounds identified in at least half of all individuals from any population. The Kovats gas chromatographic retention index (RI) is reported for each compound. Mean amounts (ng) in *H. cydno* (CYD), *H. eleuchia* (ELEU), *H. elevatus* (ELEV), *H. erato* (ERAT), *H. melpomene* (MEL), *H. sapho* (SAPH), *H. timareta* (TIM).**

| **Compound** | **RI** | **CYD** | **ELEU** | **ELEV** | **ERAT** | **MEL** | **SAPH** | **TIM** |
| --- | --- | --- | --- | --- | --- | --- | --- | --- |
| Benzaldehyde | 961 | 2.30 | 0.00 | 0.00 | 0.54 | 0.00 | 0.00 | 4.33 |
| β-Myrcene | 990 | 0.00 | 0.00 | 5.96 | 0.00 | 9.32 | 0.00 | 0.00 |
| Unknown | 1018 | 0.00 | 0.00 | 0.43 | 0.33 | 0.38 | 589.07 | 0.00 |
| Limonene | 1024 | 0.00 | 0.00 | 0.00 | 3.95 | 0.02 | 0.00 | 0.00 |
| (*Z*)-β-Ocimene | 1030 | 5.21 | 21.55 | 30.71 | 54.25 | 47.61 | 13.01 | 0.00 |
| Phenylacetaldehyde | 1036 | 0.81 | 0.00 | 0.00 | 0.85 | 0.00 | 0.00 | 0.26 |
| (*E*)-β-Ocimene | 1054 | 4.30 | 232.69 | 16663.34 | 2556.24 | 22841.08 | 26.96 | 47.32 |
| Unknown | 1076 | 0.00 | 0.00 | 0.00 | 145.11 | 0.00 | 0.00 | 0.00 |
| *o*-Guaiacol | 1090 | 1.95 | 0.13 | 0.00 | 0.13 | 0.15 | 0.00 | 0.00 |
| Nonanal | 1099 | 0.00 | 0.00 | 0.00 | 0.25 | 0.13 | 0.00 | 0.62 |
| Phenylethanol | 1109 | 0.02 | 0.00 | 0.00 | 2.92 | 0.00 | 0.00 | 0.00 |
| (3*E*,5*E*)-2,6-Dimethyl-1,3,5,7-octatetraene | 1120 | 0.00 | 0.00 | 6.89 | 0.72 | 3.53 | 0.00 | 0.00 |
| Alloocimene | 1129 | 0.00 | 0.00 | 4.03 | 0.13 | 15.00 | 0.00 | 0.00 |
| Benzyl cyanide | 1139 | 2078.02 | 0.00 | 0.00 | 210.30 | 0.00 | 0.00 | 518.81 |
| (4*E*,6*Z*)-2,6-Dimethyl-2,4,6-octatriene | 1140 | 0.00 | 0.00 | 0.00 | 0.02 | 8.36 | 0.00 | 0.00 |
| Pentyl/isopentyl 3-methylbutyrate & other unknown | 1145 | 0.00 | 0.00 | 0.00 | 0.54 | 0.00 | 0.00 | 0.00 |
| Benzyl acetate | 1165 | 0.00 | 0.00 | 0.00 | 0.37 | 0.00 | 0.32 | 0.00 |
| 2-sec-Butyl-3-methoxypyrazine | 1174 | 18.39 | 0.00 | 0.00 | 0.00 | 6.29 | 0.00 | 235.01 |
| Unknown | 1174 | 0.00 | 0.00 | 1.69 | 10.38 | 0.11 | 0.00 | 0.00 |
| Naphthalene | 1175 | 0.93 | 3.39 | 9.75 | 4.10 | 5.46 | 1.35 | 0.00 |
| 2-Methoxy-3-isobutylpyrazine | 1181 | 32.99 | 0.00 | 0.00 | 0.00 | 4.09 | 0.00 | 0.00 |
| Unknown | 1184 | 2.40 | 0.00 | 0.84 | 0.00 | 3.26 | 6.87 | 0.00 |
| Unknown | 1185 | 0.75 | 0.00 | 10.05 | 0.00 | 5.74 | 0.00 | 0.00 |
| Unknown ester | 1188 | 0.00 | 0.00 | 0.00 | 4.65 | 0.09 | 1.29 | 0.00 |
| Methyl salicylate | 1189 | 1.01 | 1.74 | 5.90 | 1.52 | 0.33 | 3.60 | 0.09 |
| Unknown | 1198 | 0.00 | 0.00 | 71.27 | 0.34 | 2.13 | 0.00 | 0.00 |
| β-Cyclocitral | 1217 | 0.00 | 0.00 | 0.00 | 8.04 | 0.18 | 0.00 | 0.00 |
| (*Z*)-3-Hexenyl isobutyrate | 1237 | 459.58 | 8.88 | 0.00 | 84.10 | 0.18 | 219.32 | 0.00 |
| Hexyl-3-methyl butyrate | 1241 | 1501.33 | 0.00 | 0.00 | 138.19 | 0.24 | 0.24 | 0.00 |
| Phenylacetaldhyde oxime | 1261 | 2.81 | 0.00 | 0.00 | 1.74 | 0.00 | 0.00 | 0.23 |
| Unknown | 1265 | 0.00 | 40.65 | 0.00 | 0.05 | 0.00 | 11.32 | 0.00 |
| Unknown | 1271 | 4.29 | 0.00 | 0.00 | 1.07 | 0.00 | 0.00 | 0.00 |
| 3-Undecanone | 1275 | 0.00 | 0.00 | 0.00 | 5212.91 | 1.08 | 0.00 | 0.00 |
| 1-Tridecene | 1286 | 0.00 | 0.00 | 0.00 | 38.85 | 0.00 | 0.00 | 0.00 |
| Dihydroedulan II | 1290 | 58.67 | 37.69 | 27.07 | 48.53 | 62.82 | 70.79 | 13.38 |
| Unknown aromatic | 1299 | 15.95 | 0.00 | 0.00 | 590.01 | 0.06 | 0.00 | 0.00 |
| Unknown | 1312 | 2.81 | 0.00 | 0.00 | 62.16 | 0.04 | 6.19 | 0.00 |
| Hexyl 3-methyl-2-butenoate | 1321 | 8.64 | 0.00 | 0.00 | 7.17 | 0.00 | 0.00 | 0.00 |
| Unknown | 1324 | 0.00 | 0.00 | 0.00 | 0.68 | 0.00 | 2.11 | 0.00 |
| Unknown | 1337 | 0.00 | 0.00 | 0.00 | 11.25 | 0.00 | 0.00 | 0.00 |
| Unknown | 1358 | 10.20 | 0.00 | 0.00 | 0.00 | 0.00 | 0.00 | 0.00 |
| Unknown | 1361 | 0.00 | 60.32 | 0.00 | 0.10 | 0.00 | 25.76 | 0.00 |
| Unknown | 1372 | 0.00 | 5.90 | 0.00 | 0.02 | 0.00 | 4.08 | 0.00 |
| α-Copaene | 1373 | 2.00 | 0.64 | 0.00 | 0.92 | 1.03 | 0.00 | 0.94 |
| (*Z*)-3-Hexenyl hexanoate | 1381 | 129.18 | 24.31 | 0.00 | 0.00 | 0.10 | 0.38 | 0.00 |
| Unknown alkene | 1382 | 0.00 | 0.00 | 0.00 | 1.07 | 0.01 | 0.00 | 0.00 |
| (*Z*)-3-Hexyl hexanoate (branched?) | 1386 | 542.38 | 884.69 | 0.00 | 0.00 | 0.00 | 197.88 | 0.00 |
| Hexyl hexanoate | 1386 | 804.85 | 1.39 | 0.00 | 0.05 | 0.01 | 0.00 | 0.00 |
| Unknown | 1390 | 8.56 | 6.42 | 0.00 | 2.12 | 4.85 | 0.00 | 37.05 |
| Hexyl hexenoate | 1390 | 1644.70 | 6.00 | 0.00 | 0.00 | 0.00 | 0.00 | 0.00 |
| Unknown ester | 1392 | 0.00 | 0.00 | 0.00 | 18.51 | 0.00 | 0.00 | 0.00 |
| Unknown | 1393 | 0.00 | 112.69 | 0.00 | 1.66 | 0.42 | 41.68 | 0.00 |
| Unknown | 1396 | 1419.45 | 736.73 | 418.50 | 236.94 | 452.03 | 0.00 | 1674.30 |
| Unknown | 1402 | 0.00 | 17.49 | 0.00 | 0.16 | 0.00 | 8.17 | 0.00 |
| Unknown | 1413 | 0.00 | 0.00 | 0.00 | 0.00 | 0.00 | 6.98 | 0.00 |
| Unknown | 1415 | 0.00 | 27.80 | 0.00 | 1.13 | 0.00 | 16.01 | 0.00 |
| β-Caryophyllene | 1417 | 4.87 | 27.33 | 0.00 | 5.34 | 3.81 | 1.34 | 0.00 |
| Unknown | 1423 | 13.27 | 0.00 | 0.00 | 0.00 | 0.00 | 0.00 | 0.00 |
| Unknown | 1424 | 0.00 | 0.00 | 0.00 | 6.39 | 0.00 | 0.00 | 0.00 |
| β-Copaene | 1427 | 0.29 | 0.00 | 0.00 | 1.42 | 0.35 | 0.00 | 0.00 |
| Unknown terpene | 1427 | 0.00 | 0.00 | 0.00 | 0.79 | 0.00 | 1.07 | 0.00 |
| Unknown aromatic | 1433 | 27.96 | 24.21 | 0.00 | 9.72 | 18.46 | 0.00 | 97.33 |
| Hexyl (*E*)-2-hexenoate | 1436 | 40.40 | 0.00 | 0.00 | 0.03 | 0.00 | 0.00 | 0.00 |
| 7,8-Dihydro-β-ionone | 1436 | 0.00 | 867.12 | 0.00 | 110.16 | 24.31 | 729.23 | 0.00 |
| Unknown monoterpene | 1438 | 151.66 | 0.00 | 0.00 | 0.00 | 0.00 | 0.00 | 0.00 |
| Unknown | 1442 | 0.31 | 0.00 | 0.00 | 2.95 | 0.00 | 0.00 | 0.00 |
| Unknown | 1444 | 0.00 | 27.03 | 0.00 | 2.23 | 0.31 | 8.16 | 0.00 |
| Unknown | 1445 | 36.34 | 0.00 | 0.00 | 0.02 | 0.00 | 0.00 | 0.00 |
| 6,10-Dimethyl-5,9-undecadien-2-one | 1449 | 0.00 | 0.00 | 0.00 | 5.79 | 0.00 | 0.00 | 0.00 |
| Alloramadendrene | 1458 | 0.00 | 0.00 | 0.00 | 1.53 | 0.32 | 0.00 | 0.00 |
| 11-Dodecanolide | 1466 | 0.90 | 0.00 | 0.00 | 0.00 | 15.09 | 0.00 | 0.00 |
| Unknown | 1468 | 0.00 | 7.53 | 0.00 | 0.00 | 0.00 | 0.13 | 0.00 |
| Unknown | 1469 | 23.42 | 0.00 | 0.44 | 0.00 | 0.00 | 0.00 | 0.00 |
| Unknown | 1470 | 34.37 | 0.00 | 0.00 | 0.43 | 0.29 | 0.00 | 0.00 |
| Unknown | 1475 | 3.50 | 0.00 | 0.00 | 0.00 | 0.00 | 0.00 | 0.00 |
| Unknown | 1478 | 7.88 | 0.00 | 0.00 | 0.00 | 0.00 | 0.00 | 0.00 |
| Unknown hexenyl ester | 1480 | 0.00 | 279.44 | 0.00 | 0.00 | 0.00 | 11.27 | 0.00 |
| Germacrene | 1486 | 4.01 | 1.79 | 0.20 | 20.33 | 6.40 | 0.87 | 0.00 |
| Unknown | 1488 | 0.16 | 0.00 | 0.00 | 1.58 | 0.00 | 0.00 | 0.00 |
| Valencene | 1492 | 2.58 | 0.00 | 0.00 | 2.06 | 0.00 | 0.00 | 0.00 |
| 5-Decanolide | 1493 | 59.26 | 0.00 | 0.00 | 0.00 | 0.00 | 0.00 | 0.00 |
| Pentadecane | 1500 | 0.00 | 0.00 | 2.22 | 1.77 | 0.67 | 0.00 | 0.00 |
| *(E*,*E*)-α-Farnesene | 1511 | 19.52 | 0.00 | 36.63 | 47.08 | 5.37 | 0.00 | 0.00 |
| 12-Dodecanolide | 1515 | 10.12 | 0.00 | 0.00 | 0.00 | 85.27 | 0.00 | 0.47 |
| δ-Cadinene | 1522 | 4.33 | 1.64 | 0.30 | 10.73 | 1.11 | 0.00 | 0.55 |
| Unknown | 1522 | 0.00 | 0.00 | 0.00 | 0.00 | 0.53 | 0.00 | 0.92 |
| Homovanillyl alcohol | 1523 | 0.00 | 0.00 | 0.14 | 0.06 | 1.33 | 0.00 | 2.86 |
| Dihydroactinidiolide | 1532 | 0.00 | 0.00 | 0.00 | 0.30 | 0.00 | 0.00 | 0.00 |
| Unknown | 1535 | 0.00 | 20.69 | 0.00 | 0.00 | 0.00 | 0.57 | 0.00 |
| Calcorene | 1540 | 0.00 | 0.00 | 0.26 | 0.06 | 0.06 | 0.00 | 0.00 |
| Mellein | 1540 | 0.00 | 0.61 | 0.00 | 170.43 | 0.02 | 0.00 | 0.00 |
| Unknown | 1543 | 0.43 | 0.14 | 0.09 | 0.01 | 0.02 | 0.00 | 1.34 |
| (*E*)-Nerolidol | 1563 | 0.86 | 24.21 | 0.51 | 5.89 | 0.28 | 0.00 | 0.00 |
| Unknown | 1565 | 0.00 | 0.00 | 0.00 | 45.28 | 0.00 | 0.00 | 0.00 |
| Ester hexanoate | 1566 | 14.57 | 0.00 | 0.00 | 0.00 | 0.06 | 0.00 | 0.00 |
| (*Z*)-3-Hexenyl benzoate | 1571 | 6.34 | 2.32 | 0.00 | 0.06 | 0.00 | 0.31 | 0.00 |
| Unknown | 1573 | 91.19 | 0.00 | 0.00 | 0.00 | 0.00 | 0.00 | 0.00 |
| Unknown | 1576 | 3.31 | 0.00 | 0.00 | 0.00 | 0.00 | 0.00 | 0.00 |
| Unknown | 1581 | 0.00 | 0.00 | 0.00 | 0.00 | 0.08 | 0.00 | 14.63 |
| Unknown alkene | 1581 | 0.75 | 0.00 | 0.00 | 0.73 | 0.00 | 0.00 | 0.00 |
| Unknown | 1583 | 31.61 | 0.00 | 0.00 | 0.00 | 0.00 | 0.00 | 0.00 |
| Hexadecane | 1600 | 0.00 | 0.00 | 5.43 | 2.94 | 1.30 | 0.00 | 0.00 |
| Unknown | 1607 | 0.00 | 0.00 | 0.00 | 0.00 | 4.17 | 0.00 | 0.00 |
| 1-*epi*-Cubenol | 1608 | 0.00 | 0.00 | 0.00 | 0.57 | 0.01 | 0.00 | 0.00 |
| Unknown | 1621 | 0.00 | 0.00 | 0.00 | 17.27 | 0.00 | 0.00 | 0.00 |
| Unknown | 1625 | 2.54 | 5.39 | 0.00 | 0.00 | 0.00 | 0.00 | 0.00 |
| Unknown | 1630 | 24.71 | 0.00 | 0.00 | 0.00 | 0.00 | 0.00 | 0.00 |
| Unknown | 1632 | 4.56 | 0.00 | 0.00 | 0.00 | 0.00 | 0.00 | 0.00 |
| Unknown | 1636 | 1.01 | 0.00 | 4.74 | 0.00 | 3.85 | 0.00 | 0.00 |
| Unknown | 1639 | 0.00 | 0.00 | 2.69 | 1.01 | 0.61 | 0.00 | 0.00 |
| Unknown | 1643 | 3.59 | 0.00 | 0.00 | 0.00 | 0.00 | 0.00 | 0.00 |
| δ-Cadinol | 1644 | 0.36 | 0.37 | 0.00 | 23.23 | 0.14 | 0.00 | 0.00 |
| Unknown | 1646 | 0.00 | 0.00 | 0.00 | 0.00 | 16.03 | 0.00 | 0.00 |
| α-Cadinol | 1652 | 0.00 | 0.00 | 0.00 | 2.21 | 0.00 | 0.00 | 0.00 |
| Unknown | 1656 | 0.34 | 0.68 | 6.80 | 1.45 | 0.83 | 0.99 | 0.00 |
| Unknown | 1661 | 0.00 | 8.51 | 0.00 | 0.00 | 0.00 | 1.22 | 0.00 |
| Heptadecene | 1679 | 0.00 | 0.00 | 1.25 | 1.02 | 0.21 | 0.00 | 0.00 |
| Pentadecanolide | 1691 | 0.00 | 412.12 | 0.00 | 0.25 | 0.00 | 211.71 | 0.00 |
| Heptadecane | 1700 | 0.00 | 0.00 | 2.18 | 1.03 | 0.33 | 0.00 | 0.00 |
| Unknown1 | 1704 | 0.00 | 0.00 | 0.00 | 0.00 | 16.03 | 0.00 | 0.00 |
| Unknown2 | 1704 | 0.00 | 0.00 | 0.00 | 0.94 | 0.00 | 0.00 | 0.00 |
| Unknown macrolide | 1714 | 0.00 | 0.00 | 0.00 | 23.77 | 0.00 | 0.00 | 0.00 |
| 14-Tetradecanolide | 1733 | 67.79 | 0.00 | 12.28 | 0.00 | 157.47 | 0.00 | 20.83 |
| Unknown | 1750 | 0.96 | 0.00 | 0.00 | 1048.23 | 0.01 | 0.00 | 0.00 |
| Unknown hydrocarbon | 1750 | 0.92 | 0.99 | 4.22 | 14.93 | 1.85 | 1.08 | 1.29 |
| Unknown | 1754 | 9.36 | 0.00 | 0.00 | 0.00 | 0.00 | 0.00 | 0.00 |
| Tetradecanoic acid | 1755 | 0.13 | 0.00 | 0.00 | 32.14 | 0.00 | 0.00 | 0.00 |
| Ethyl benzoate | 1762 | 0.33 | 0.00 | 0.38 | 0.49 | 0.48 | 0.00 | 0.37 |
| Unknown ester | 1765 | 39.52 | 0.00 | 0.00 | 0.00 | 0.00 | 0.00 | 0.00 |
| Unknown | 1769 | 0.65 | 0.00 | 0.00 | 1.57 | 0.00 | 0.00 | 0.00 |
| Unknown | 1771 | 0.00 | 5.11 | 0.00 | 0.00 | 0.00 | 0.00 | 0.00 |
| Unknown hexenyl or cyclo pentyl ester | 1775 | 797.54 | 4.61 | 0.00 | 0.00 | 2.75 | 0.00 | 78.44 |
| Unknown macrolide | 1777 | 264.35 | 0.00 | 0.00 | 0.00 | 0.00 | 0.00 | 1.85 |
| Unknown | 1787 | 0.00 | 0.00 | 0.00 | 2.58 | 0.00 | 0.00 | 0.00 |
| Octadecane | 1800 | 0.00 | 0.00 | 2.34 | 0.75 | 0.45 | 0.00 | 0.00 |
| Unknown | 1802 | 0.00 | 0.00 | 0.00 | 1.16 | 0.00 | 0.00 | 0.00 |
| Hexadecadienolide-15-olide | 1807 | 0.00 | 0.00 | 0.00 | 28.73 | 0.00 | 0.00 | 0.00 |
| Unknown | 1817 | 0.25 | 6.33 | 0.00 | 0.00 | 0.00 | 0.00 | 0.50 |
| Hexadecen-11-olide | 1828 | 108.76 | 0.00 | 0.00 | 0.00 | 0.00 | 0.00 | 7.50 |
| Unknown | 1833 | 0.00 | 0.00 | 0.00 | 8.79 | 0.00 | 0.00 | 0.00 |
| Hexadecenolide | 1835 | 0.00 | 0.00 | 0.00 | 0.00 | 0.21 | 0.00 | 1.49 |
| Neophytadiene | 1836 | 0.00 | 315.81 | 0.00 | 0.13 | 0.00 | 21.27 | 0.00 |
| Hexadecenolide | 1845 | 100.43 | 0.00 | 0.00 | 0.00 | 0.00 | 0.00 | 0.00 |
| Unknown | 1846 | 29.45 | 10.05 | 0.48 | 3.94 | 9.70 | 36.17 | 1.66 |
| 15-Hexadecanolide | 1853 | 17.17 | 0.00 | 0.29 | 0.05 | 4.48 | 0.00 | 10.29 |
| Hexanyl ester | 1853 | 0.00 | 1489.91 | 0.00 | 0.00 | 0.25 | 150.00 | 0.00 |
| Hexadecenolide | 1854 | 24.29 | 0.00 | 0.00 | 0.00 | 0.00 | 0.00 | 0.20 |
| Unknown | 1856 | 21.35 | 0.00 | 0.00 | 0.00 | 1.40 | 0.00 | 5.16 |
| Unknown | 1860 | 6.31 | 0.00 | 0.00 | 0.00 | 2.13 | 0.00 | 13.14 |
| Hexadecenolide | 1861 | 441.07 | 0.00 | 7.61 | 0.00 | 3.61 | 0.00 | 63.47 |
| Neophytadiene | 1861 | 0.00 | 74.61 | 0.00 | 0.00 | 0.00 | 4.33 | 0.00 |
| Benzyl salicylate | 1870 | 1.36 | 6106.41 | 23.37 | 34.69 | 17.95 | 3322.00 | 0.00 |
| Nonadecene | 1877 | 1.08 | 0.00 | 1.38 | 0.84 | 0.13 | 0.00 | 0.00 |
| Unknown macrolide | 1878 | 0.00 | 54.84 | 0.00 | 0.00 | 0.00 | 1.08 | 0.00 |
| Neophytadiene | 1878 | 0.00 | 117.30 | 0.00 | 0.00 | 0.00 | 7.72 | 0.00 |
| 1-Hexadecanol | 1884 | 0.00 | 0.00 | 0.00 | 11.29 | 0.21 | 0.00 | 0.00 |
| Hexadecatrienolide | 1884 | 11.33 | 0.00 | 0.00 | 0.00 | 0.00 | 0.00 | 1.06 |
| 9,11-Hexadecadien-11-olide | 1893 | 115.51 | 0.00 | 0.00 | 0.00 | 0.00 | 0.00 | 2.63 |
| Nonadecane | 1900 | 51.07 | 0.00 | 25.48 | 0.30 | 10.28 | 0.00 | 28.87 |
| Unknown | 1901 | 1.47 | 0.00 | 4.28 | 1.67 | 0.01 | 0.00 | 2.92 |
| Unknown sesquiterpene | 1902 | 0.00 | 0.00 | 39.74 | 0.00 | 12.54 | 0.00 | 0.00 |
| Unknown | 1907 | 0.00 | 0.00 | 0.00 | 18.45 | 0.00 | 0.00 | 0.00 |
| Unknown | 1915 | 0.00 | 0.00 | 2.92 | 1.86 | 0.66 | 0.00 | 0.00 |
| 16-Hexadecanolide | 1936 | 277.89 | 19.71 | 0.00 | 0.00 | 0.56 | 50.67 | 119.28 |
| Hexadecen-16-olide | 1937 | 8.02 | 1.06 | 0.00 | 0.00 | 0.50 | 0.00 | 2.24 |
| Hexyl dodecanoate | 1960 | 0.00 | 0.00 | 0.00 | 28.05 | 0.00 | 0.00 | 0.00 |
| Hexadecanoic acid | 1960 | 14.57 | 2.09 | 0.00 | 30.03 | 6.44 | 0.00 | 2.22 |
| Unknown hydrocarbon | 1962 | 0.14 | 0.00 | 0.86 | 0.23 | 0.41 | 0.00 | 0.16 |
| Unknown | 1968 | 0.17 | 0.00 | 4.11 | 1.04 | 4.14 | 0.00 | 2.49 |
| Unknown | 1970 | 0.00 | 0.00 | 0.00 | 110.51 | 0.09 | 0.00 | 0.00 |
| Brassicalactone | 1971 | 131.03 | 0.00 | 7.17 | 0.00 | 0.12 | 0.00 | 147.59 |
| Unknown | 1977 | 0.00 | 0.00 | 0.00 | 4.41 | 0.02 | 0.00 | 0.00 |
| Octadecatrienolide | 1987 | 21.38 | 0.00 | 0.00 | 0.00 | 0.00 | 0.00 | 15.07 |
| Icosane | 2000 | 25.98 | 0.00 | 25.91 | 0.02 | 36.62 | 0.00 | 41.62 |
| Octadecen-11-olide | 2008 | 183.49 | 0.00 | 0.00 | 0.00 | 0.00 | 0.00 | 57.35 |
| Unknown | 2017 | 0.00 | 0.00 | 0.00 | 101.74 | 0.00 | 0.00 | 0.00 |
| Octadecadienolide | 2019 | 119.80 | 18.57 | 4.53 | 0.00 | 90.17 | 0.00 | 738.77 |
| Isopropyl palmitate | 2020 | 34.95 | 0.00 | 0.00 | 0.06 | 0.00 | 0.00 | 62.12 |
| Octadecadienolide | 2033 | 45.14 | 1.93 | 0.00 | 0.00 | 12.42 | 1.94 | 1318.41 |
| Unknown pentyl ester | 2033 | 0.00 | 0.00 | 0.00 | 226.39 | 0.00 | 0.00 | 0.00 |
| (*Z*)-9-Octadecen-11-olide | 2038 | 8513.77 | 0.00 | 99.69 | 0.00 | 0.74 | 0.00 | 3257.65 |
| 2-Phenylethyl decanoate | 2038 | 0.33 | 0.00 | 0.00 | 10.75 | 0.00 | 0.00 | 0.00 |
| (*Z*)-9-Octadecen-13-olide | 2044 | 5137.74 | 0.00 | 53.09 | 0.00 | 56.84 | 0.00 | 5916.34 |
| Octadecanoic acid ester | 2045 | 105.19 | 0.00 | 0.00 | 0.00 | 8.67 | 0.00 | 39.54 |
| Unknown macrolide | 2048 | 15.61 | 0.00 | 7.30 | 0.00 | 12.89 | 0.00 | 93.84 |
| 12-Octadecanolide | 2055 | 119.92 | 0.00 | 0.00 | 0.00 | 0.00 | 0.00 | 12.06 |
| Unknown | 2055 | 0.00 | 0.00 | 0.00 | 5.68 | 0.00 | 0.00 | 0.00 |
| (*Z*9,*E*11,*Z*15)-9,11,15- Octadecatrien-13-olide | 2061 | 2354.90 | 0.00 | 0.00 | 0.00 | 0.41 | 0.00 | 614.24 |
| Unknown | 2062 | 107.29 | 0.00 | 0.00 | 0.00 | 0.00 | 0.00 | 0.00 |
| (*E*)-Octadec-9-en-12-olide | 2065 | 429.44 | 0.00 | 0.00 | 0.00 | 0.00 | 0.00 | 98.09 |
| (*Z*9,*E*11)- 9,11-Octadecadien -13-olide | 2065 | 22006.74 | 0.63 | 8.26 | 0.00 | 0.96 | 0.00 | 1063.22 |
| Henicosene | 2068 | 12.36 | 4.66 | 1313.29 | 6.98 | 65.72 | 0.00 | 7.26 |
| Henicosene | 2072 | 110.06 | 0.00 | 2430.30 | 53.95 | 149.32 | 9.61 | 100.88 |
| Henicosene | 2086 | 3.92 | 14.05 | 19.45 | 3.03 | 24.45 | 0.00 | 8.53 |
| Unknown | 2093 | 0.00 | 0.00 | 0.00 | 6.63 | 0.00 | 0.00 | 0.00 |
| Octadecadienolide | 2095 | 786.47 | 0.00 | 0.00 | 0.00 | 0.00 | 0.00 | 7.97 |
| Henicosane | 2100 | 1925.11 | 0.00 | 2265.48 | 19.39 | 3803.86 | 0.00 | 4272.72 |
| Unknown terpene ester | 2120 | 0.00 | 0.00 | 0.00 | 52.68 | 0.00 | 0.00 | 0.00 |
| 9-Octadecen-18-olide | 2123 | 1114.19 | 10.97 | 48.20 | 0.06 | 25.86 | 18.23 | 1703.38 |
| 18-Octadecanolide | 2135 | 532.01 | 3.25 | 0.00 | 52.57 | 12.35 | 0.00 | 217.04 |
| Unknown terpene ester | 2139 | 0.00 | 0.00 | 0.00 | 714.11 | 0.00 | 0.00 | 0.00 |
| Unknown | 2144 | 24.10 | 0.00 | 0.00 | 0.00 | 0.04 | 0.00 | 32.41 |
| Unknown terpene ester | 2150 | 0.00 | 0.00 | 0.00 | 2490.83 | 0.47 | 0.00 | 0.00 |
| Unknown amide | 2157 | 0.05 | 0.00 | 0.00 | 1.94 | 0.28 | 0.00 | 1.03 |
| Ethyl oleate | 2159 | 6.32 | 0.00 | 103.74 | 0.00 | 74.15 | 0.00 | 656.14 |
| Unknown | 2160 | 0.16 | 0.00 | 0.00 | 18.63 | 0.70 | 0.00 | 0.00 |
| Octadecanoic acid | 2161 | 5.98 | 0.00 | 0.00 | 1.57 | 1.78 | 0.00 | 2.31 |
| Unknown | 2166 | 0.16 | 0.00 | 0.00 | 246.29 | 0.29 | 0.00 | 0.00 |
| Unknown | 2168 | 0.00 | 0.00 | 0.00 | 56.48 | 0.05 | 0.00 | 27.60 |
| Isopentyl octadecadienoate | 2177 | 24.35 | 0.00 | 55.57 | 0.00 | 6.99 | 0.00 | 44.03 |
| Octadecadienolide | 2178 | 416.33 | 0.00 | 0.00 | 0.00 | 0.71 | 0.00 | 416.42 |
| Butyl hexadecanoate | 2186 | 0.00 | 0.00 | 0.00 | 1.52 | 48.32 | 0.00 | 395.49 |
| Isopropyl oleate | 2188 | 6455.01 | 0.00 | 536.83 | 0.00 | 247.13 | 0.00 | 9353.76 |
| Docosane | 2200 | 0.47 | 0.00 | 15.50 | 1.89 | 42.63 | 0.00 | 13.07 |
| Unknown diterpene | 2205 | 0.00 | 0.00 | 0.00 | 326.23 | 0.00 | 0.00 | 0.00 |
| Unknown macrolide | 2210 | 46.31 | 0.00 | 0.00 | 0.00 | 0.00 | 0.00 | 30.66 |
| Icosen-13-olide | 2218 | 715.91 | 0.00 | 1.38 | 0.00 | 0.00 | 0.00 | 76.47 |
| Icosanal | 2219 | 43.01 | 1.31 | 12.38 | 12.68 | 5.65 | 0.00 | 14.32 |
| Isopropyl octadecanoate | 2224 | 242.19 | 0.00 | 2.52 | 0.00 | 0.80 | 0.00 | 187.79 |
| Unknown | 2229 | 0.00 | 0.00 | 0.00 | 2.16 | 0.00 | 0.00 | 0.00 |
| Unknown | 2238 | 105.25 | 0.00 | 0.00 | 0.00 | 2.50 | 0.00 | 178.84 |
| Isopropyl octadecadienolate | 2241 | 2165.26 | 0.00 | 9.06 | 0.00 | 0.00 | 0.00 | 1339.28 |
| 2-Phenylethyl dodecanoate | 2243 | 0.00 | 0.00 | 0.00 | 335.57 | 0.00 | 0.00 | 0.00 |
| Unknown | 2245 | 0.00 | 0.00 | 0.00 | 0.00 | 5.88 | 0.00 | 156.00 |
| Icosadien-15-olide | 2250 | 312.24 | 0.00 | 0.00 | 0.00 | 0.00 | 0.00 | 5.33 |
| Isoprenyl palmitate | 2254 | 0.55 | 0.00 | 0.00 | 5.47 | 0.63 | 0.00 | 0.00 |
| Icosenol | 2255 | 0.00 | 0.00 | 732.36 | 0.11 | 4.68 | 0.00 | 0.00 |
| Unknown | 2258 | 0.00 | 0.00 | 0.00 | 5.14 | 0.00 | 0.00 | 0.00 |
| 13-Icosanolide | 2259 | 67.71 | 0.00 | 0.00 | 0.00 | 0.00 | 0.00 | 100.04 |
| Isopropyl 9,12-octadecadienoate | 2263 | 673.31 | 0.00 | 0.00 | 0.00 | 0.00 | 0.00 | 101.38 |
| Unknown | 2263 | 0.00 | 0.00 | 0.00 | 0.00 | 0.00 | 0.00 | 747.20 |
| Tricosene | 2270 | 383.95 | 2.22 | 898.75 | 21.47 | 152.97 | 7.71 | 64.87 |
| Tricosene | 2275 | 387.50 | 30.92 | 3461.39 | 223.41 | 268.81 | 36.72 | 247.60 |
| Unknown | 2279 | 0.00 | 0.00 | 0.00 | 106.05 | 0.00 | 0.00 | 0.00 |
| Unknown | 2280 | 0.00 | 0.00 | 0.00 | 3.03 | 0.00 | 0.00 | 0.00 |
| Isopentyl ester | 2285 | 0.00 | 0.00 | 34.49 | 0.00 | 5.54 | 0.00 | 1.71 |
| Isobutyl oleate | 2287 | 67.84 | 0.00 | 843.45 | 0.00 | 231.52 | 0.00 | 147.86 |
| Unknown | 2290 | 3.67 | 0.66 | 4.14 | 0.07 | 0.99 | 29.37 | 1.56 |
| Unknown | 2297 | 0.00 | 0.00 | 0.00 | 26.19 | 0.00 | 0.00 | 0.00 |
| Tricosane | 2300 | 110.63 | 4.50 | 125.94 | 56.30 | 355.85 | 0.41 | 229.22 |
| Unknown | 2305 | 47.55 | 0.00 | 43.17 | 0.00 | 77.52 | 0.00 | 453.19 |
| Unknown terpene ester | 2310 | 0.00 | 0.00 | 0.00 | 13314.02 | 6.20 | 0.00 | 0.91 |
| Unknown | 2320 | 66.77 | 0.00 | 0.00 | 0.00 | 0.00 | 0.00 | 0.00 |
| Unknown | 2320 | 0.00 | 0.00 | 0.00 | 29.16 | 0.00 | 0.00 | 0.00 |
| Unknown | 2324 | 0.00 | 0.00 | 0.00 | 278.45 | 0.00 | 0.00 | 0.00 |
| Unknown | 2325 | 60.06 | 0.00 | 1.16 | 0.00 | 0.49 | 0.00 | 36.57 |
| Unknown | 2329 | 0.00 | 0.00 | 0.00 | 37.54 | 0.00 | 0.00 | 0.00 |
| 20-Icosanolide | 2330 | 264.60 | 0.00 | 0.00 | 0.00 | 3.32 | 0.00 | 333.62 |
| Unknown | 2336 | 0.00 | 0.00 | 0.00 | 13.06 | 0.00 | 0.00 | 0.00 |
| Unknown amide | 2347 | 28.54 | 0.31 | 53.14 | 110.07 | 28.27 | 8.05 | 87.41 |
| Docosadienolide | 2347 | 33.41 | 0.00 | 0.00 | 0.00 | 0.14 | 0.00 | 25.95 |
| Unknown | 2349 | 0.00 | 0.00 | 0.00 | 7.66 | 0.00 | 0.00 | 0.00 |
| Hexyl hexadecenoate | 2351 | 36.26 | 0.00 | 0.00 | 0.47 | 0.96 | 0.00 | 0.00 |
| Hexyl hexadecenoate | 2353 | 66.90 | 5.49 | 0.00 | 14.14 | 8.16 | 0.00 | 0.00 |
| Butyl octadecadienoate | 2355 | 14.13 | 0.00 | 6.38 | 0.00 | 51.78 | 0.00 | 130.87 |
| Unknown | 2360 | 18.01 | 0.00 | 0.00 | 0.00 | 0.00 | 0.00 | 23.06 |
| Butyl oleate | 2361 | 0.00 | 0.00 | 74.72 | 18.11 | 1176.37 | 0.00 | 14387.66 |
| Unknown sesterterpene hydrocarbon | 2370 | 0.00 | 0.00 | 0.00 | 463.94 | 0.00 | 0.00 | 0.00 |
| Unknown | 2373 | 0.00 | 0.00 | 0.00 | 1.12 | 0.00 | 0.00 | 0.00 |
| (*Z*)-3-Hexenyl hexadecanoate | 2379 | 253.15 | 39.69 | 1.10 | 163.00 | 119.56 | 1.55 | 50.99 |
| Butyl octadecanoate | 2386 | 0.00 | 0.00 | 0.00 | 0.13 | 29.30 | 0.00 | 233.43 |
| Unknown | 2387 | 0.00 | 0.00 | 0.00 | 82.89 | 0.00 | 0.00 | 0.00 |
| Unknown macrolide | 2392 | 115.24 | 0.00 | 1.91 | 0.00 | 0.00 | 0.00 | 3.73 |
| Unknown | 2396 | 0.00 | 0.00 | 0.00 | 0.00 | 0.67 | 0.00 | 2287.35 |
| Unknown | 2398 | 0.00 | 0.00 | 0.00 | 27.11 | 0.00 | 0.00 | 0.00 |
| Tetracosane | 2400 | 0.25 | 0.00 | 14.73 | 12.99 | 10.04 | 0.00 | 0.81 |
| Icosyl acetate | 2408 | 0.00 | 0.00 | 0.00 | 53.34 | 0.00 | 0.00 | 0.00 |
| 2-Phenylethyl tetradecenoate | 2415 | 0.00 | 0.00 | 0.00 | 7.07 | 0.00 | 0.00 | 0.00 |
| Unknown | 2418 | 80.06 | 0.00 | 0.00 | 39.32 | 2.59 | 0.00 | 236.97 |
| 19-Methylicosyl acetate | 2422 | 73.80 | 0.46 | 5.47 | 5.31 | 9.16 | 0.00 | 0.00 |
| Unknown sesterterpene hydrocarbon | 2427 | 0.00 | 0.00 | 0.00 | 195.86 | 0.00 | 0.00 | 0.00 |
| Unknown | 2432 | 0.00 | 0.00 | 0.00 | 3.35 | 0.00 | 0.00 | 0.00 |
| Unknown terpene ester | 2435 | 0.00 | 0.00 | 0.00 | 1458.48 | 0.00 | 0.00 | 0.00 |
| Isoprenyl octadec-11-enoate | 2436 | 10.90 | 0.00 | 5.22 | 36.11 | 12.59 | 0.00 | 16.40 |
| Unknown | 2451 | 0.00 | 0.00 | 0.00 | 871.50 | 0.00 | 0.00 | 0.00 |
| Unknown | 2453 | 56.78 | 0.00 | 0.00 | 0.00 | 0.00 | 0.00 | 25.51 |
| Docosenolide | 2455 | 27.89 | 0.00 | 0.00 | 0.00 | 0.00 | 0.00 | 61.64 |
| Unknown | 2455 | 9.18 | 0.00 | 0.00 | 0.16 | 5.35 | 0.00 | 11.35 |
| Unknown | 2458 | 0.00 | 0.00 | 0.00 | 15.53 | 0.00 | 0.00 | 0.00 |
| (Z)-13-Docosen-1-ol | 2460 | 74.44 | 4.12 | 1423.15 | 2.06 | 66.81 | 0.00 | 46.98 |
| Unknown | 2460 | 0.00 | 0.00 | 0.00 | 14.00 | 2.34 | 0.00 | 0.00 |
| Unknown | 2465 | 8.67 | 1.07 | 0.00 | 0.00 | 1.91 | 0.00 | 6.27 |
| Unknown | 2467 | 12.46 | 0.00 | 3.47 | 0.00 | 0.71 | 12.63 | 0.00 |
| Unknown | 2472 | 66.43 | 0.00 | 47.08 | 29.73 | 224.34 | 0.00 | 431.21 |
| Icosenolide | 2475 | 59.95 | 0.00 | 0.00 | 0.00 | 0.00 | 0.00 | 105.64 |
| Unknown | 2487 | 0.00 | 0.00 | 0.00 | 375.56 | 0.00 | 0.00 | 0.00 |
| 1-Docosanol | 2490 | 1606.60 | 26.14 | 2019.66 | 333.96 | 864.97 | 26.49 | 817.09 |
| Unknown | 2490 | 0.00 | 0.00 | 0.00 | 15.96 | 0.00 | 0.00 | 0.00 |
| Unknown terpene ester | 2494 | 0.00 | 0.00 | 0.00 | 1352.10 | 0.00 | 0.00 | 0.00 |
| Pentacosane | 2500 | 32.26 | 67.62 | 89.86 | 127.99 | 128.42 | 9.12 | 115.21 |
| Unknown | 2503 | 0.00 | 0.00 | 0.00 | 4.98 | 0.00 | 0.00 | 0.00 |
| Unknown aromatic ester | 2511 | 0.00 | 0.00 | 0.00 | 128.44 | 0.00 | 0.00 | 0.00 |
| Unknown | 2523 | 0.00 | 0.00 | 0.00 | 237.75 | 0.00 | 0.00 | 0.00 |
| 11-Methylpentacosane | 2532 | 0.00 | 0.92 | 281.90 | 0.00 | 42.18 | 0.00 | 10.23 |
| Hexyl octadecadienoate | 2544 | 0.00 | 0.00 | 0.00 | 0.00 | 0.00 | 0.00 | 110.64 |
| Hexyl octadecanoate | 2550 | 50.62 | 182.27 | 1.10 | 55.23 | 97.19 | 29.26 | 40.25 |
| Docosen-22-olide | 2551 | 294.37 | 0.00 | 0.00 | 0.00 | 4.41 | 0.00 | 162.16 |
| Hexyl octadecenoate & (*Z*)-3-Hexenyl octadecenoate | 2557 | 7351.86 | 894.88 | 283.47 | 719.73 | 975.62 | 233.40 | 4569.69 |
| Hexenyl octadecatrienoate & (*Z*)-3-Hexenyl octadecatrienoate | 2561 | 1040.52 | 101.96 | 0.00 | 35.29 | 226.38 | 7.71 | 438.91 |
| Benzyl hexadecanoate | 2569 | 64.43 | 0.00 | 0.00 | 9.76 | 0.00 | 0.00 | 46.40 |
| (*Z*)-3-Hexenyl octadecanoate | 2581 | 194.51 | 4.37 | 2.36 | 16.92 | 82.54 | 0.66 | 26.03 |
| Unknown | 2588 | 0.00 | 0.00 | 0.00 | 163.90 | 0.00 | 0.00 | 0.00 |
| Hexyl octadecanoate | 2590 | 1304.10 | 0.00 | 0.00 | 0.00 | 0.00 | 0.00 | 417.94 |
| Unknown | 2593 | 0.00 | 0.00 | 0.00 | 402.39 | 0.00 | 0.00 | 0.00 |
| Hexacosane | 2600 | 0.00 | 7.30 | 14.07 | 27.73 | 20.78 | 0.69 | 25.86 |
| 1,3-Docosanediol (cyclic dImethylsilyl derivative) | 2604 | 626.77 | 5.10 | 132.85 | 144.33 | 148.81 | 1.31 | 135.78 |
| Hexyl octadecanoate | 2621 | 113.97 | 0.00 | 0.00 | 0.00 | 0.62 | 60.76 | 386.24 |
| Unknown | 2626 | 0.00 | 0.00 | 5.05 | 0.00 | 2.25 | 0.00 | 0.00 |
| Unknown | 2628 | 0.00 | 0.00 | 0.00 | 58.75 | 0.00 | 0.00 | 0.00 |
| Unknown | 2631 | 0.00 | 22.63 | 0.52 | 3.16 | 0.01 | 0.18 | 0.00 |
| Unknown sesterterpene | 2636 | 0.00 | 0.00 | 0.00 | 5844.44 | 2.26 | 0.00 | 0.00 |
| Unknown | 2636 | 1.81 | 0.00 | 0.00 | 19.29 | 0.00 | 0.00 | 0.00 |
| Unknown | 2655 | 0.00 | 0.00 | 24.45 | 9.13 | 2.33 | 0.00 | 50.19 |
| Unknown | 2656 | 0.00 | 0.00 | 0.00 | 1.64 | 12.89 | 0.00 | 0.00 |
| Unknown | 2657 | 4.70 | 0.00 | 0.00 | 500.69 | 0.00 | 0.00 | 0.00 |
| Tetracosenol | 2664 | 2831.00 | 60.76 | 1491.35 | 747.98 | 1024.14 | 41.48 | 1536.26 |
| Unknown | 2670 | 0.00 | 0.00 | 3.92 | 37.23 | 3.22 | 0.00 | 0.00 |
| Heptacosene | 2675 | 18.98 | 4.46 | 20.89 | 38.51 | 85.04 | 0.00 | 168.58 |
| Unknown | 2690 | 91.66 | 0.00 | 0.00 | 0.00 | 0.00 | 0.00 | 0.00 |
| Unknown | 2694 | 1345.00 | 0.00 | 0.00 | 177.23 | 171.59 | 0.00 | 478.60 |
| 1-Tetracosanol | 2694 | 631.83 | 11.12 | 1574.08 | 801.80 | 806.30 | 13.84 | 578.68 |
| Unknown | 2699 | 0.00 | 0.00 | 0.00 | 436.84 | 0.00 | 0.00 | 0.00 |
| Heptacosane | 2700 | 323.89 | 237.58 | 238.02 | 222.08 | 280.33 | 139.08 | 318.56 |
| 1,3-Tricosanediol (cyclic dImethylsilyl derivative) | 2704 | 99.89 | 0.00 | 0.00 | 0.00 | 0.00 | 0.00 | 0.00 |
| Unknown aromatic ester | 2718 | 0.00 | 0.00 | 0.00 | 65.07 | 0.00 | 0.00 | 0.00 |
| 11-Methylheptacosane | 2730 | 0.00 | 48.91 | 42.01 | 0.00 | 28.38 | 2.56 | 65.08 |
| Unknown | 2733 | 5.38 | 14.02 | 0.00 | 62.42 | 0.00 | 0.00 | 37.76 |
| Unknown | 2735 | 0.00 | 0.00 | 0.00 | 489.73 | 0.00 | 0.00 | 0.00 |
| Unknown2 | 2735 | 60.84 | 0.00 | 0.00 | 0.00 | 0.00 | 0.00 | 0.00 |
| Cholestadiene | 2744 | 32.82 | 7.42 | 1.03 | 6.29 | 7.37 | 0.00 | 17.60 |
| Unknown | 2746 | 0.00 | 0.00 | 2.07 | 0.31 | 1.29 | 0.00 | 0.00 |
| Tetracosenolide | 2749 | 1191.60 | 0.00 | 21.22 | 0.00 | 7.38 | 0.00 | 80.97 |
| Unknown | 2753 | 32.55 | 59.64 | 20.13 | 2.30 | 19.50 | 44.22 | 31.60 |
| Unknown terpene | 2755 | 0.00 | 0.00 | 0.00 | 1585.67 | 0.19 | 0.00 | 0.00 |
| 13-Docosenamide | 2770 | 171.59 | 3.47 | 297.50 | 442.28 | 190.84 | 88.54 | 408.92 |
| Unknown | 2783 | 163.02 | 0.00 | 32.86 | 3.66 | 42.87 | 0.00 | 54.20 |
| 1,3-Tetracosanediol (cyclic dImethylsilyl derivative) | 2799 | 302.17 | 1.02 | 229.22 | 155.63 | 149.58 | 0.00 | 87.12 |
| Octacosane | 2800 | 0.00 | 0.49 | 14.21 | 41.69 | 16.16 | 0.00 | 41.03 |
| Unknown triterpene | 2823 | 5.39 | 0.00 | 303.06 | 6449.52 | 46.90 | 0.96 | 19.88 |
| Hexacosanal | 2829 | 1270.51 | 6.38 | 120.75 | 759.60 | 200.84 | 0.72 | 124.36 |
| Unknown | 2840 | 27.15 | 0.00 | 0.00 | 1083.40 | 0.00 | 0.00 | 14.53 |
| Unknown | 2855 | 10.50 | 0.00 | 52.75 | 6.24 | 2.43 | 0.00 | 32.95 |
| Unknown | 2864 | 23.12 | 0.00 | 0.00 | 99.22 | 0.00 | 0.00 | 1.25 |
| Cholesta-3,5-diene | 2871 | 24.12 | 7.15 | 0.52 | 8.31 | 11.48 | 0.00 | 11.90 |
| Unknown | 2891 | 52.16 | 0.00 | 84.04 | 172.51 | 30.87 | 0.00 | 30.63 |
| Unknown triterpene | 2891 | 0.00 | 0.00 | 0.00 | 3126.08 | 0.99 | 0.00 | 0.00 |
| Unknown | 2898 | 376.41 | 0.00 | 28.91 | 54.49 | 186.54 | 0.00 | 123.42 |
